# Laminar and turbulent flow effects in high-pressure homogenization of liposomes and perfluorocarbon nanoemulsions

**DOI:** 10.1038/s41598-024-78550-9

**Published:** 2024-11-13

**Authors:** Larissa J. Lubitz, Harden Rieger, Gero Leneweit

**Affiliations:** 1 ABNOBA GmbH, 75223 Niefern-Öschelbronn, Germany; 2 Carl Gustav Carus-Institute, 75223 Niefern-Öschelbronn, Germany; 3 Institute of Mechanical Process Engineering and Mechanics, Karlsruhe Institute of Technology, 76131 Karlsruhe, Germany

**Keywords:** Biomedical engineering, Drug development

## Abstract

Since flow characteristics are still largely unexplored for high-pressure homogenization, we investigated particle break-up at different Reynolds numbers and transition ranges in two channels (Y- and Z-channel). While the channel geometries are often treated as “black boxes”, opening the channels and measuring their geometries allowed a detailed analysis of flow conditions. Transitions from laminar to turbulent flow for pressures of 250–2,000 bar have measurable effects on the sizes of perfluorocarbon (PFC)-nanoemulsion droplets emulsified by phospholipids processed simultaneously in liposomal conformation. Laminar flow has a higher size-reducing rate with growing pressure compared to turbulent flow and leads to a minimum in polydispersity. A density-driven sucrose gradient allows differential analysis of size-reducing effects on liposomes and PFC-nanoemulsion droplets separately. Liposomes can be broken up in both laminar and turbulent flow at the same size reduction rate. In contrast, emulsion droplets have much smaller size reduction rates in turbulent flow and need sufficient emulsifiers, made available by liposomal break-up, to enable size decreases. Repetitive homogenization is only effective for a limited number of cycles. Beyond this threshold, size distributions remain similar or can be deteriorated because of increased particle collisions and aggregation or coalescence effects.

**Supplementary Information:**

The online version contains supplementary material available at 10.1038/s41598-024-78550-9.

## Introduction

High-pressure homogenization is used in industry for many decades to optimize the product characteristics of foods, beverages and pharmaceuticals. Multiple mechanical effects were studied^1–6^ and also summarized in different reviews^7–9^. However, the complex interplay of channel flows with particles in the micro- and nanometer size ranges and their effects on the dispersed phases and emulsifiers leave many questions still open. Specifically, the effect of the Reynolds number and the transition from laminar to turbulent flows is largely unexplored, while recent studies disclose various aspects of cavitation in droplet break-up^10,11^.

The focus of our study is on the flow conditions and their effects on two particle species which are interconnected: a perfluorocarbon-in-water emulsion and liposomes consisting of natural phospholipids which act as emulsifiers for the hydrophobic phase. Phospholipids and liposomes were chosen as a model system due to their widespread use as biocompatible emulsifiers, formulation components and delivery systems for active ingredients in foods and pharmaceuticals. However, phospholipids can form organogels with most natural lipids such as e.g., triglycerides in water^12^, which may lead to unstable phospholipid emulsion systems^13^. Therefore, we selected a hydrophobic excipient that avoids any interaction with phospholipids. This is among the many advantages known for perfluorocarbons (PFC) that become increasingly important excipients for pharmaceutical and medical applications as nanoemulsions. For example, they are utilized for ^19^F MRI, taking advantage of the absence of fluorine in human organisms on the one hand and the appropriate nuclear magnetic resonance (NMR) properties on the other^14^. Historically, PFC emulsions were used as artificial blood substitutes, as perfluorocarbons have an enormous gas-binding capacity^15,16^. Fluosol^®^ and Oxygent™ are among the emulsions formerly approved by the FDA and were used in retinal surgery, for example^17–20^.

In addition to the use of PFC nanoemulsions as pharmaceutical end products, they can also be used as intermediates for the production of asymmetric liposomes in which the inner and outer bilayers have a different, i.e. asymmetric composition. Based on the mechanism published by Träuble and Grell in 1971, Pautot et al. 2003 already demonstrated to produce asymmetric liposomes by centrifuging a water-in-oil emulsion into a water phase enriched with phospholipids^21,22^. However, the resulting vesicles in the range of 1 to 10 μm are not applicable in pharmaceutical applications.

In contrast to symmetric liposomes, the production of asymmetric liposomes offers the advantage of considerably increasing the encapsulation efficiency. Encapsulation efficiencies of high molecular weight molecules are below 50% with conventional production methods for liposomes, such as the film method or high-pressure homogenization^23–26^. Ullmann et al. 2021 were able to show that an encapsulation efficiency of more than 90% ca. be achieved with liposomes based on a water-in-perfluorocarbon nanoemulsion^27^.

When perfluorocarbons are added to a liposomal suspension for emulsification, the phospholipids distribute in two different conformations: the monolayer state emulsifying the PFCdroplets and the liposomal bilayer state. In order to separate the two particle species – liposomes and nanoemulsion droplets – after their formation, a sucrose gradient can be used. The application of this analytical separation method not only isolates liposomes from PFC emulsion droplets, but also produces fractions of very narrow size distributions to improve the quality of the size measurements. This density gradient method enables a size separation of the PFC-droplets, since the droplets’ masses increase with *r*^3^ of their droplet radius *r* while their friction only increases linearly with *r*. The separation and size distributions are achieved by density differences of liposomes and PFC-droplets on the one hand and size differences between emulsion droplets on the other hand in the centrifugal field. This is due to the density of the PFC used, which is about twice that of water. In addition to particle size and flow velocity, the product temperature was also of interest, as Grapentin et al. 2015 were able to show that high temperatures (heat sterilization) result in high amounts of liposomes in PFC/W emulsions^28^. Moreover, visualizations of PFC nanoemulsions emulsified by liposomes have been provided by Grapentin et al.^28^ under almost identical processing conditions as studied here. This imaging can be referred to as an additional source of visual evidence for the PFC nanoemulsions under the conditions used in the current study.

Regarding the mechanical technologies to produce homogeneously dispersed phases, there is a variety of methods for producing such nanoemulsions. These can primarily be classified into high-energy and low-energy methods^29,30^. In the high-energy methods, strong disruptive forces are generated, which ensure the break-up of large droplets. These include high-pressure valve homogenization, microfluidization, and ultrasonication^29,31,32^. In the case of high-pressure valve homogenization, the emulsion is forced through a narrow orifice, where forces are acting on the dispersed droplets such as the fluctuating strains of turbulence, laminar shear forces and the peak pressures of collapsing cavitation bubbles. All of these mechanical deformations can lead to the break-up of droplets.

In the present study, we used microfluidization in which the emulsion is pressed through one or several microchannels in series. Here, we studied a so-called Y-channel followed by a Z-channel (see Fig. 2). The Y-channel includes a T-shaped split into two parallel channels which are re-unified downstream, thereby forming an interaction chamber allowing collisions in case of particles being driven to a stagnation point due to their inertia. Thus, particle collision can be another class of forces besides normal and shear strains and the collapse jets in cavitation bubbles, all of them potentially contributing to droplet break-up.

In order to estimate the flow conditions in the different segments of the Y- and Z-channels, we estimated the Reynolds numbers of each segment based on the measured flow rates for each pressure condition at the channel entry. As the channels’ cross-sections are either circular or rectangular for the microfluidic Y- and Z-channel geometries (see Fig. 2), we only needed to consider the fundamental flow conditions for these two basic geometries. Concerning the circular channel cross-section, Rotta (1959) was able to prove experimentally that the laminar-turbulent transition occurs around the Reynolds number *Re* ≈ 2,300^33^. However, the stability of laminar circular tube flow is extremely sensitive to the suppression of fluctuations such as enhanced filtration of inflow conditions, dampening of vibrations and extremely smooth wall surfaces. Several authors could prove that the Reynolds number for the laminar-turbulent transition can be as high as *Re* > 100,000 for extremely low amplitudes of disturbances (volume flux Q of disturbance Q_dist_ < 10^− 4^ Q_flow_ of the main flow Q_flow_, see Peixinho (2007)^34^. Eckhardt (2008) showed that this is a consequence of the non-linearity of circular pipe flow, but in the current context we will refer to the transition Reynolds number found by Rotta (1959) since both the microfluidic channel walls and the inflow conditions will not enable small amplitudes of perturbation^33,35^.

Concerning channel flow in an infinite slit flow, the transition from laminar to turbulent flows was solved analytically by Orszag (1971) using the Orr-Sommerfeld equation, which is based on the assumption of a linear instability of the Navier-Stokes equations^36^. Orszag showed the onset of linear instability to occur for *Re* > 5,772. For flows through rectangular channels of different aspect ratios α, Chang et al. (2012) used an energy gradient method to calculate the theoretical boundary for the onset of the laminar-turbulent transition 1,250 < *Re*_*crit, theor.*_ < 2,700^37^. On the other side, Chen et al. (2007) and others found experimentally critical Reynolds numbers *Re*_*crit, exp.*_ which were 5–35% higher, showing a discrepancy between the onset of small fluctuations and their measurability^38^.

To pursue the focus of our study, we will unravel the effects the flow conditions on liposomes and emulsion droplets by tackling the following research questions: What are the effects of the homogenization pressure on the flow rates in microfluidic channels? Are the transitions from laminar to turbulent channel flows detectable in any flow parameters or particle sizes? Will the flow conditions act differentially on the particle species (i.e. liposomes or emulsion droplets) for the different size fractions? Will the number of cycles through the microfluidic channel system have unlimited effects on particle sizes or will it be limited after a finite number of cycles?

## Materials and methods

### Materials

 Purified egg yolk phospholipid was obtained as a commercial mixture (Lipoid E80, Lipoid GmbH, Ludwigshafen) containing ~ 80% phosphatidylcholine. Monosodium phosphate dihydrate (NaH_2_PO_4_ × 2 H_2_O), disodium phosphate dihydrate (Na_2_HPO_4_ × 2 H_2_O), D(+)-saccharose, hydrochloric acid (37% fuming), and cholesterol were obtained from Carl Roth GmbH & Co. KG (Karlsruhe, Germany) and of pharmaceutical quality according to the European Pharmacopoeia. Perfluoroperhydrophenanthrene was obtained from F2 Chemicals Ltd. (Preston, Lancashire, United Kingdom). A summary of the relevant physicochemical properties of Perfluoroperhydrophenanthrene can be found in Table 1.

**Table 1:** Summary of different physicochemical properties of Perfluoroperhydrophenanthrene.

Name	Boiling point [°C]	Density ρ at 25 °C [g/mL]	Dynamic viscosity η at 25 °C [mPa s]	Structure
Perfluoroperhydrophenanthrene	215	2.03	28.4	

### Preparation of buffer and sucrose solutions

 The buffer used for emulsion preparation consisted of 10 mM phosphate (6.08 mM Na_2_HPO_4_ × 2 H_2_O and 3.92 mM NaH_2_PO_4_ × 2 H_2_O) without adjustment for isotonicity, and the pH was adjusted to 7.4 with 1 M hydrochloric acid. The buffer was used sterile-filtered and degassed for all manufacturing steps. The sucrose solutions (20%, 30%, 40%, 50% and 60% w/v) were prepared by weighing the required amount of sucrose by addition of the corresponding volume of deionised water and subsequent filtration through a 0.45 μm syringe filter (Carl Roth GmbH & Co. KG, Karlsruhe, Germany). Figure S1 in the Supplements shows the verification of the gradient for the separation of liposomes using a sucrose gradient.

### Preparation of nanoemulsions

 For the preparation of the PFC/W nanoemulsions, a lipid film with a concentration of 150 mM consisting of E80/cholesterol in the ratio 95:5 mol% was prepared in the first step using the thin-film method. After rehydration of the lipid film with buffer to prepare a lipid stock solution of 150 mM, dilution to 7.5 mM was performed for each emulsion batch for further processing. Pre-mixing of the lipid vesicles contained in the buffer was performed by high shear mixing using a rotary shear-mixer (Ultra-Turrax^®^ T25 easy clean equipped with the dispersing tool S25 EC–T–C–18, Ika^®^-Werk GmbH & Co. KG, Staufen im Breisgau, Germany) at 8,000 rpm for 5 min. Subsequently, the pre-emulsion was prepared by adding 2.5% (v/v) Perfluoroperhydrophenanthrene and mixing again using the Ultra-Turrax^®^ at 15,000 rpm for 10 min. Immediately afterwards, the pre-emulsion was transferred into a syringe for further processing on the Microfluidizer LV1 (Microfluidics, Westwood, USA), which was used with a combination of two interaction chambers (F12Y 75 μm and H20Z 200 μm). The emulsion was homogenized for 6 cycles at different homogenization pressures ranging from 250 to 2000 bar, with cooling of the process housing from 1000 bar onwards. For *N*_*c*_-dependent experiments, the emulsions were prepared at 1000 bar with cooling of the process housing. Cooling was not used for the series of tests to determine the pressure-dependent volume flow and the emulsion temperature. To prevent lipid oxidation, all process steps of the emulsion production using Ultra-Turrax^®^ and Microfluidizer LV1 were carried out using an argon atmosphere. A stopwatch was used to measure the stroke time, and an infrared thermometer was used to measure the product temperature at the outlet.

### Dynamic light scattering (DLS)

 The mean intensity-weighted hydrodynamic diameter (Z-Average) of the PFC droplets of the prepared PFC/W nanoemulsion was determined by dynamic light scattering (DLS) on the ZetaSizer Nano ZS90 (Malvern Panalytical, Malvern, United Kingdom). The measurement was carried out at different times during processing, after final production, and after separation by means of a sucrose gradient. The measurement was carried out at 25 °C and a scattering angle of 90°. The particle size is presented as the so-called Z-averaged hydrodynamic diameter (Z-Ave), and the width of the particle size distribution is expressed as the polydispersity index (PdI). All measurements were carried out in triplicate. The values are given as the mean of the three independent samples being produced, where each sample is measured for 3 measurements with each 5 runs.

### Separation using the sucrose gradient

**Fig. 1:**
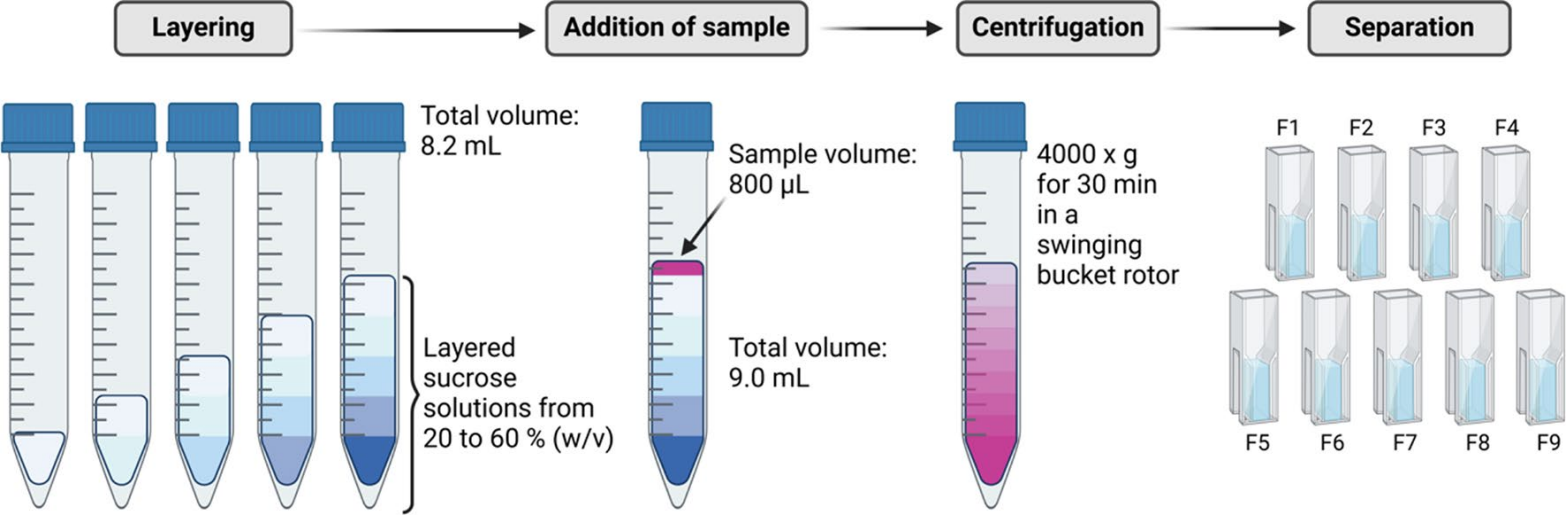
Schematic representation of the procedure of the density-based separation of the PFC/W nanoemulsion using the sucrose gradient. The sample is layered on top and centrifuged after layering the sucrose solutions in ascending concentration. Finally, the sample is separated into 9 fractions of 1 mL each starting from the top and characterized using DLS.

 For analytical separation of the remaining liposomes and separation of the PFC-droplets according to their size, a sucrose gradient was performed. For this purpose, sucrose solutions were layered on top of each other in ascending concentration by underlaying in a 15 mL tube to a total volume of 8.2 mL. Subsequently, 0.8 mL of the PFC/W nanoemulsion was layered dropwise on top and centrifuged for 30 min at 4,000 x g in the swinging-bucket rotor. Subsequently, 9 fractions of 1 mL each were removed stepwise from the top and transferred directly into a polystyrene disposable semi-micro cuvette (ROTILABO^®^, Carl Roth GmbH & Co. KG, Karlsruhe, Germany) for subsequent characterization by dynamic light scattering. Figure 1 shows a schematic scheme of the procedure.

### Channel imaging and drawings

 Channel photographs were taken using Canon EOS 7D (Tokyo, Japan) equipped with a SIGMA DC 18–250 mm 1–3.5–6.1 Macro HSM (SIGMA Deutschland GmbH, Rödermark, Germany). Drawings of the channels were prepared using the software Autodesk Inventor 2017 (Autodesk GmbH, München, Germany).

### Data presentation and statistical analysis

All experiments were performed in triplicate and data were reported as arithmetic mean with standard deviation. For the statistical evaluation of the data, a single factor analysis of variance (ANOVA) was performed at a significance level of *p* > 0.05 followed by a two-sample t-test assuming equal variances. All analysis was performed using Microsoft Excel (Reymond, WA, USA).

## Results

### The geometry of the interaction chambers and calculation of Reynolds numbers

**Fig. 2:**
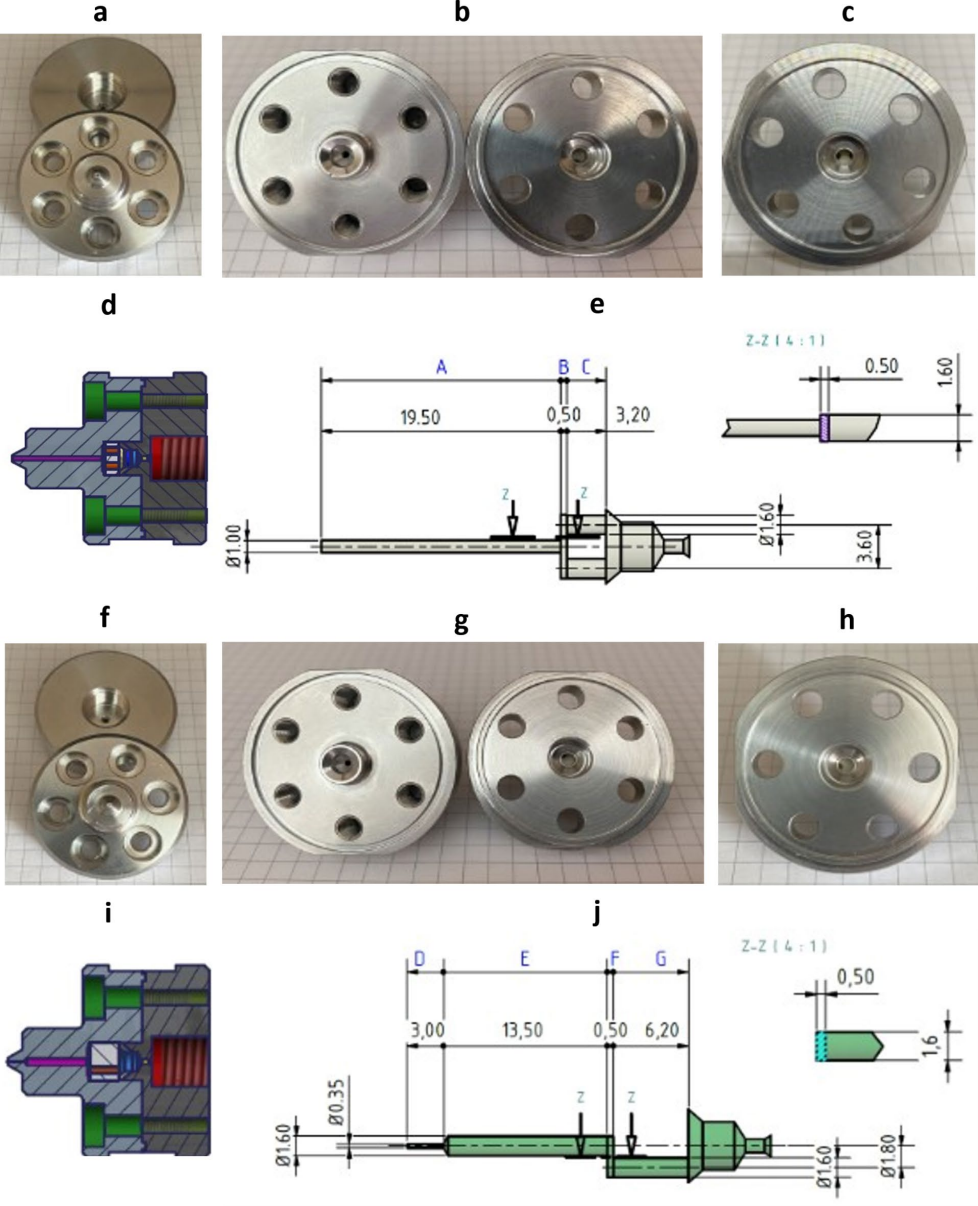
Photographs and CAD drawings of the opened Y- and Z-channel types with the interaction chambers inside. (**a**–**c**) Pictures of the Y-channel of type F12Y, (**f**–**h**) pictures of the Z-channel of type H20Z, where **a** and **h** show the outer surfaces of the channels, **b** and **g** show the inner surfaces of the channels, and **c** and **h** show a detailed view of both channels focused on the ceramic disc therein. (**d** and **e**) Drawings of the Y-channel of type F12Y, (**i** and **j**) drawings of the Z-channel of type H20Z, where **e** and **j** show a detailed drawing of the dimensions of the Y-channel (**e**) and Z-channel (**j**).

In order to obtain precise knowledge of the channel geometry, the high-pressure homogenization channels were opened, dimensioned and CAD drawings were designed for illustration purposes. The two image sections of Fig. 2a and f show the inlet orifice of the Y- and Z-channel, respectively. Surprisingly, the inlet is smaller for the Z-channel (Fig. 2f and j section D) than for the Y-channel (Fig. 2a and e section A), although the Z-channel width is specified as 200 μm according to the manufacturer. The Y-channel is specified with a channel width of 75 μm. This can also be seen in Fig. 2a and f respectively, as the inlet holes in the channel housings in the lower part of the photographs are different. As the Fig. 2b and g show the inner surfaces of the channel housing, differences in the off-centred holes in the right parts of both figures can be seen. Figure 2c and h show this channel housing parts in more detail. Figure 2c shows two off-centred, laterally symmetric holes in the ceramic disc (left and right) which are characteristic for the Y-channel, whereas Fig. 2h shows only one off-centred hole in the ceramic disc, characteristic for the Z-channel (left). Figure 2d shows that the inlet to the interaction chamber is significantly larger for the Y-channel compared to Fig. 2i for the Z-channel. In addition, when comparing Fig. 2e and j (detailed drawing of the channel dimensions), it can be seen that the volume flow for the Y-channel is divided into two parallel tubular channels (Fig. 2e section C) re-uniting in the interaction chamber.

## The effect of pressure on homogenization of mixed liposomes and emulsions

### Pressure-dependent particle size

A pre-emulsion consisting of the perfluorocarbon (PFC, 2.5% v/v) in a phosphate-buffered phospholipid/cholesterol suspension (E80/cholesterol 95:5 mol%, 7.5 mM) was prepared by a rotor-stator mixer (15,000 rpm, 10 min). This pre-emulsion was stable enough to be used for the feed of the high-pressure homogenizer without phase separation during influx since high-pressure homogenizers cannot be used with two separated phases in their injection system. However, the parameters used for pre-emulsification only avoided phase separation during influx, but the pre-emulsion was not stable enough to be characterized in size by DLS because of rapid disintegration and separation. The described procedure was chosen with the intention to keep the effect of rotor-stator shear to a minimum and allow high-pressure homogenization to have an almost exclusive effect on droplet break-up during each passage through the homogenization chamber, from now on referred to as ‘cycle’. The minimum number of cycles chosen for the emulsification via high-pressure homogenization was 6 cycles since only after this minimum of accumulated passage time a size distribution stable for > 48 h could be achieved. Thus, the conditions used for emulsification were the minimum requirements to characterize the effect of pressure on the size distribution of a PFC stabilized by phospholipids.

**Fig. 3:**
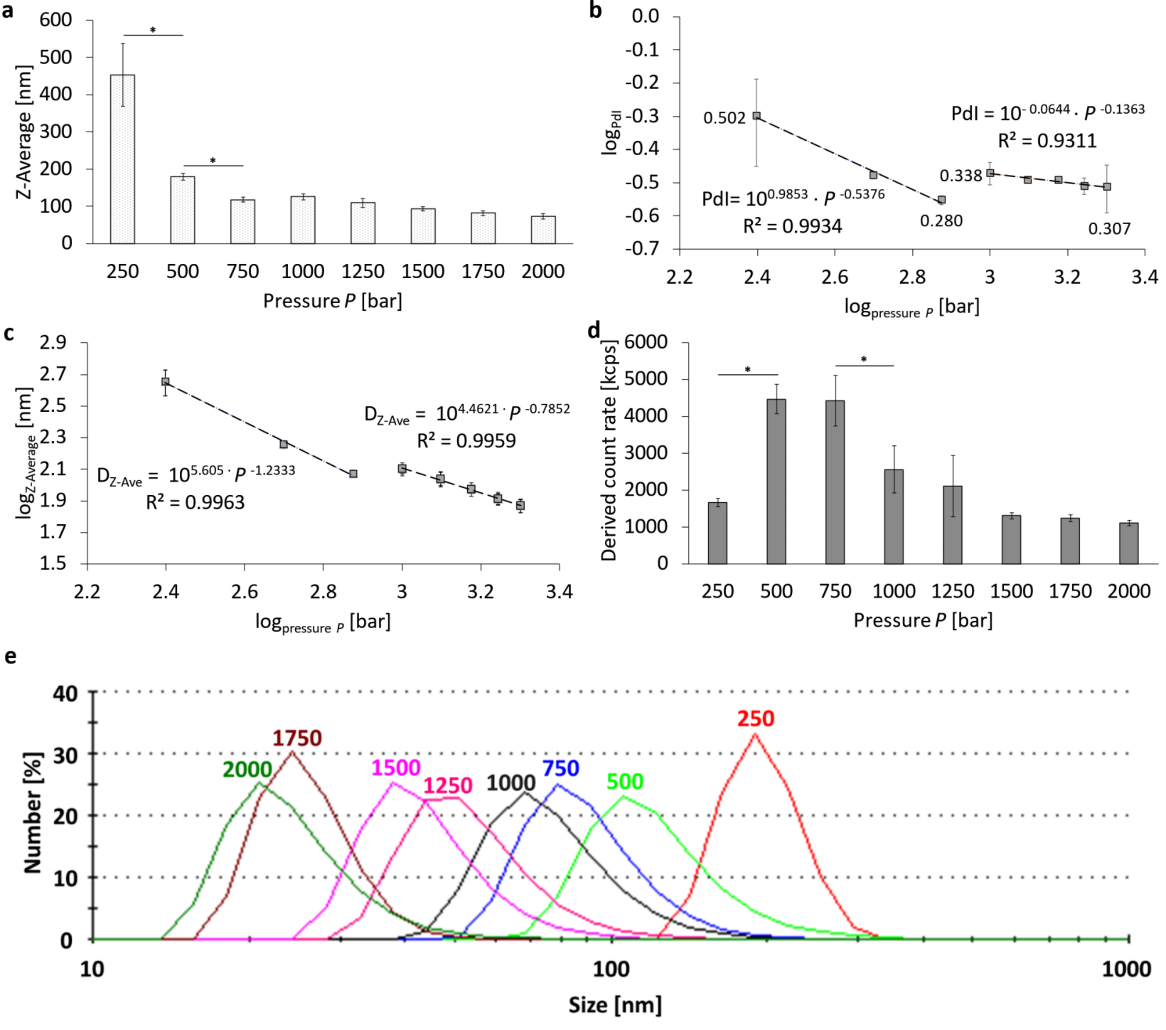
Pressure-dependent characteristics of a PFC/W nanoemulsion after 6 cycles of high-pressure homogenization using the Y- and Z-channel combined in series. Representation of (**a**) the Z-Average (Z-Ave) at linear scale, (**b**) the logarithmic representation of the polydispersity index (PdI) with PdI values next to the grey squares for 250, 750, 1,000 and 2,000 bar with power-law regressions in two regimes (250–750 bar and 1,000–2,000 bar), (**c**) logarithmic representation of the Z-Ave with power-law regressions in two regimes (250–750 bar and 1,000–2,000 bar), (**d**) Derived Count Rates (DCR) at linear scale, (**e**) representation of the individual size distribution as plot of the number % against the particle size. The bars or grey squares represent the mean values with the standard deviation for *n* = 3 as number of independent samples. A one-way ANOVA followed by a two-sample t-test was performed with a significance level (signed by *) for an error probability *p* < 0.05.

Figure 3 summarizes the particle size, the corresponding PdI and the Derived Count Rates (DCR) of a PFC/W nanoemulsion processed for 6 cycles at different homogenization pressures. The largest Z-averaged particle size, from now on called ‘Z-Ave’, is at the lowest pressure 250 bar with 452.2 nm ± 84.2 (Fig. 3a). The smallest particle size is achieved after processing at 2,000 bar with a Z-Average of 73.7 nm ± 6.8 nm. Comparing Fig. 3a and c it becomes apparent that the linear bar plot in Fig. 3a does not fully reveal significant correlations in the resulting particle sizes, only in the lower pressure range *P* ≤ 750 bar significant differences can be seen between data points. The pressure-dependent PdI values (Fig. 3b) are also highest at 250 bar with PdI = 0.502 ± 0.148. However, the minimum PdI is not found at 2,000 bar, but at a pressure of 750 bar with 0.280 ± 0.009. Moreover, it is clearly visible that the PdI values decrease in two different regimes of homogenization pressures *P*: in the lower pressure range *P* ≤ 750 bar the PdI decreases with a power-law of PdI ~ $1/\sqrt{P}$ while in the second regime for *P* ≥ 1,000 bar only a very small decrease of PdI with increasing pressure takes place.

Figure 3d shows a significant increase of the DCR when increasing the homogenization pressure from 250 bar to 500 bar and a significant decrease from 750 to 1,000 bar. To better visualize and quantify the decay of droplet sizes at higher pressures, the Z-Ave is also presented in a logarithmic plot in Fig. 3c. It clearly reveals two regimes of homogenization, but instead of a minimum, it shows a transition in a plateau range between 750 and 1,000 bar regarding the size at 750 bar. Logarithmic regressions for the two regimes show a power-law of for the Z-averaged droplet sizes *D*_*Z−Ave*_ ~ *P*^− 1.233^ for the regime I (*P* ≤ 750 bar) and *D*_*Z−Ave*_ ~ *P*^− 0.7852^ for regime II (*P* ≥ 1,000 bar). This comparison shows that the decrease in Z-Ave with increasing pressure is considerably stronger in the lower-pressure regime I than in the higher-pressure regime II.

As shown in Fig. 3e, an increase in the homogenization pressure shifts the particle distribution also very clearly to lower size ranges when presented in terms of number percentages. This means that liposomal disintegration and nanoemulsion droplet atomization not only take place for the larger particle fractions, but equally for the smaller fractions.

In order to elucidate the mechanisms behind the transition from regime I to II, the Reynolds numbers *Re* are assessed for the different sections of the microfluidic channels causing high-pressure drops by frictional energy dissipation to discriminate ranges of laminar flows and their transition to turbulence. This needs an estimate of the mean flow velocity ${\bar{u}}_{i}$ at the cross-sectional area ${A}_{i}$ where the subscript *i* = A, B, or C for the Y-channel and D, E, F or G for the Z-channel indicates the cross-section as shown in Fig. 2e and j deduced from the total volume flow rate *Q*. This volume flow rate *Q* is an average in time by dividing the stroke volume *V* by the total duration *Δt* of the outflow:

1a and 1b $$\bar{u}_{i}=\frac{Q}{{A}_{i}}\;\; {\text{with}}\;\; Q=\frac{V}{\varDelta t}$$

The Reynolds number *Re*_*i*_ at a specific cross section of area *A*_*i*_ is defined by a characteristic length *D*_*i*_ which is either the tube diameter if the channel is circular or the hydraulic diameter for a rectangular channel with a smaller side *a* and the longer side *b*, and where ν is the kinematic viscosity. So, *Re*_*i*_ and the hydraulic diameters *D*_*B*_ and *D*_*F*_ are defined by:

2a and 2b $${Re}_{i}=\frac{{\bar{u}}_{i}\cdot {D}_{i}}{\nu}\;\; {\text{with}} \;\; {D}_{B}={D}_{F}=\frac{2ab}{a+b} {\text{for the rectangular channels}}$$

Since the manufacturer’s manual does not provide a precise geometry of the microfluidic channels used, we opened both the Y- and the Z-channel and display photographs and precisely measured channel dimensions, see Fig. 2. In order to estimate the Reynolds numbers and the according flow regimes in the complex channel geometry, we used an integrative approach by measuring the total duration of the outlet flow from the instant of the hydraulic pressure discharge. The flow rates displayed in Table 2 are both a spatial and temporal average flowing through a complex microfluidic channel geometry.

**Table 2:** Reynolds numbers *re* in the Y-channel and Z-channel at specific cross section displayed in Fig. 2e and j, respectively. The Reynolds numbers are given as arithmetic mean based on the volume flow with its standard deviation; volume flows were measured via the duration of the outlet flow in *n* = 3 independent measurements. Reynolds numbers in bold indicate transition ranges from laminar to turbulent flow in the respective channel section.

Pressure *P* [bar]	Volume Flow [mL/s]	Re_A_ at ∅ A	Re_B_ at ∅ B	Re_C_ at ∅ C	Re_D_ at ∅ D	Re_E, G_ at ∅ E & G	Re_F_ at ∅ F
250	1.85 ± 0.01	2,358	882	737	6,737	1,474	1,764
500	3.22 ± 0.2	4,100	1,533	1,281	11,714	2,562	3,067
750	4.43 ± 0.25	5,645	2,111	1,764	16,128	3,528	4,222
1,000	4.36 ± 0.01	5,549	2,075	1,734	15,855	3,468	4,151
1,250	4.99 ± 0.21	6,349	2,374	1,984	18,139	3,968	4,749
1,500	5.90 ± 0.38	7,514	2,810	2,348	21,469	4,696	5,621
1,750	6.14 ± 0.57	7,822	2,925	2,444	22,348	4,889	5,851
2,000	7.66 ± 0.24	9,752	3,647	3,048	27,864	6,095	7,295

The Reynolds numbers at the circular cross-sectional area A are > 2,300 for all pressures measured (250–2,000 bar). This means that for cross-section A only for 250 bar the flow is in a transitional stage between the laminar and turbulent regime considering classical experimental fluid dynamics^33^, while for all higher pressures the flow is fully turbulent in this tubular segment.

Section B is a channel segment with a rectangular cross-section where the influx is split at a T-junction, causing a split of the volume flow by half. This results in a drop of the Reynolds number range between 882 (at *P* = 250 bar) and 3,647 (at *P* = 2,000 bar). For a rectangular channel with cross-section B having an aspect ratio α = a/b = 0.5 mm/1.6 mm = 0.3125, a theoretical analysis by Chang et al.^37^ finds the transition Reynolds number to be *Re*_*crit, B*_ = 1,679. However, Chen et al. show experimentally for α = 0.333 that the critical Reynolds number is *Re*_*crit, exp.*_ = 2,528^38^. This is in accordance with most other experimental results for rectangular channels who find the critical Reynolds number for the onset of a laminar instability to be 5–35% higher than the theoretical analysis by Chang et al.^37^. This discrepancy is not a contradiction, since it is connected to the fact that unstable fluctuations *u’* of the linear velocity *U* are not always detected experimentally in the velocity field *u = U + u’* when the fluctuation amplitude is still small or it would need much longer channels for the convergence of experimental and theoretical findings. Based on this background, it can be concluded that in Fig. 3c the distinction of two independent regimes I and II in the Z-averaged droplet size is caused by the transition from a laminar regime I to a turbulent regime II taking place in section B of the channel geometry.

This interpretation on the physical causes of different modes in droplet homogenization implies that re-laminarization takes place when the turbulent flow from section A impinges as a circular jet at the T-junction forming the entrance of the rectangular channel section B. Experimental measurements by Sano & Tamai (2016) and theoretical analyses by Kaewbumrung & Plengsa-Ard (2024) show that re-laminarization of turbulence in a rectangular flow or at an impinging jet is realistic^39,40^. In these cases, turbulent inflow is relaminarized by the subsequent flow field in case that the latter is governed by laminar flow conditions. But why should the droplet size stop decreasing in the transition range between laminar and turbulent flow? An obvious reason is that the shear rates stop increasing and the friction factor ceases to decrease in the transition range between laminar and turbulent flow^39^. These correlations thus provide clear indications on the mechanisms of droplet break-up in high-pressure homogenization.

**Fig. 4:**
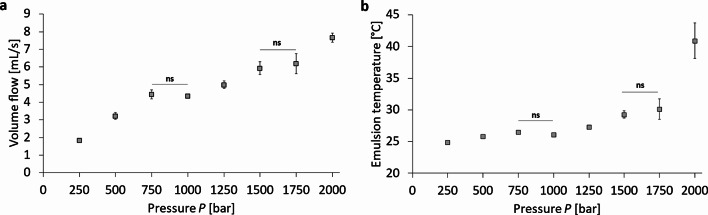
Pressure-dependent volume flow in high-pressure homogenization using the Y- and Z-channel combined in series. Representation of the pressure-dependent (**a**) volume flow and (**b**) the temperature increase of the processed fluids in an uncooled setup of the high-pressure homogenization for a PFC/W nanoemulsion. The dots represent the mean values with the standard deviation as error bars. A one-way ANOVA followed by a two-sample t-test assuming equal variances was performed to show a significance level *p* < 0.05 in all neighbouring data points except for those indicated as ‘non-significant’ (ns); *n* = 3.

Following further the pathway of the high-pressure channel, section C also shows a transition range with a corresponding Reynolds number *Re*_*C*_ = 2,348 at 1,500 bar and *Re*_*C*_ = 2,444 at 1,750 bar in Table 2. However, there are no obvious phenomena in Fig. 3 that would indicate any transitional effects on Z-averaged droplet sizes or PdI in the pressure range of 1,500 to 1,750 bar. This is different when studying the increase of the flow rate with increasing pressure in Fig. 4a and the temperature increase in Fig. 4b measured by infrared thermometry at the outflow of the emulsion from the high-pressure Y- and Z-channels used. Both diagrams show an interruption of the steady increase between 750 and 1,000 bar and between 1,500 and 1,750 bar. Correlating these facts with the Reynolds number increases in the different sections reveals that there is a quantitative correlation with the laminar-turbulent transitions in: (i) section B (for *P* between 750 and 1,000 bar) and (ii) section C (for *P* between 1,500 and 1,750 bar). Moreover, there is also a clear correlation between Fig. 4a and b, which is not self-evident since both figures are based on independent measurements (time duration of outflow vs. temperature of outflow): On the one hand, the volume flow in Fig. 4a stops increasing measurably most likely due to a transitional effect of the flow morphology in a specific section of the pathway, at lower *Q* in section B and at higher *Q* in section C. On the other hand, the temperature increase is interrupted at the same transition ranges as is the flow rate *Q*. This means that when there is also no increase in the fluid transport per unit time, there is an unchanged conductive heat transfer from the fluid volume, in which frictional energy dissipation takes place to the stainless steel surrounding of the high-pressure channels. However, flow rate and temperature *T* do not increase completely proportional in the full pressure range as can be seen in the high-pressure range with a very strong increase of *T* at 2,000 bar.

Analyzing the Reynolds numbers *Re* in the Z-channel (H20Z), it can be found that they are all in the regime of turbulence for all segments D-G and all pressures, except for the circular segments E and G at 250 bar, please see Table 2. Transition to turbulence takes place between 250 and 500 bar where *Re*_*E, G*_ equals 1,474 and 2,562, respectively. However, the laminar shear rate is not very high in these two segments before transition to turbulence. Their maximal wall shear rate is around 9,200 s^− 1^ – much lower than the shear rate in the pre-emulsifying by the rotary shear mixer (19,300 s^− 1^) and the shear rate in the Y-channel’s segment B before the onset of turbulence (49,800 s^− 1^). Therefore, the laminar-turbulent transitions in the Z-channel do not have a measurable effect on particle homogenization in the studied parameter range. This comparison shows that for a laminar turbulent transition to become effective, also the shear rate must be in an elevated range in the laminar flow regime. Due to the linear shear gradient in laminar flow and the nonlinear shear gradient in the turbulent flow range, volume-averaged shear is more effective in the laminar range compared to the turbulent range (see Discussion section). Another consequence elucidated by the comparison of the flow patterns in the Y- and Z-channels is relevant: only the serial arrangement of the Y- and Z-channel causes an enhanced friction and pressure drop in both channels. This higher dissipation shifts the transitions to turbulence to the higher pressure range, in our case between 750 and 1,000 bar (segment B) and 1,500–1,750 bar (segment C). This combination of both flow-induced (shear) and pressure-induced effects (cavitation, vibrations and elasticities of both solid channels and high pressure-induced compressible fluids) cause the homogenization which is presented here. To enable a comparison for droplet break-up with current literature^41,42^, the Weber number and Capillary number are shown in Supplementary Table S1. As can be seen in Table S1, the range for the Weber number *We*, formed by the Z-averaged particle diameter *D* and the minimum interfacial tension at a DPPC-cholesterol covered water/PPHP interface^43^ is in an extremely low range of 0.1 ≤ *We* ≤ 0.29. The capillary numbers *Ca* are in the range of 2.74 ≤ *Ca* ≤ 11.33 which is relatively high owing to the high dynamic viscosity of PPHP.

## Preparative separation of particle species

In order to separate the liposomes that were added as emulsifiers for the perfluorocarbon-in-water emulsion, a density-driven sucrose gradient (20–60% w/v) was used. For its use, 0.8 mL of sample was added as top layer above 8.2 mL of the sucrose gradient. After centrifugation (4,000 × g; 30 min), the fractions F1 to F9 were removed by slow and careful pipette aspiration from top to bottom. Figure S1 in the Supplements shows a test run for the separation of liposomes labeled with Lissamine™ Rhodamine DHPE (0.1 mol%) without addition of a perfluorocarbon phase. Here, only fractions F1 (82.8%), F2 (11.8%) and F9 (5.4%) contain the total recovery of the fluorescently labeled phospholipids. The fact that fraction 9 contains liposomes is interpreted as an artefact resulting from the aspiration technique applied to remove the different fractions by pipetting, leaving a thin wall layer which is finally aspirated together with fraction 9. Based on this background, the following analyses are undertaken and interpreted in the way that fractions 1 and 2 contain liposomes while fraction 9 is not interpreted quantitatively as it not only contains some small contamination by liposomes but being the collection basin which is contaminated by the wall layers of all fractions F1 – F8.

**Fig. 5:**
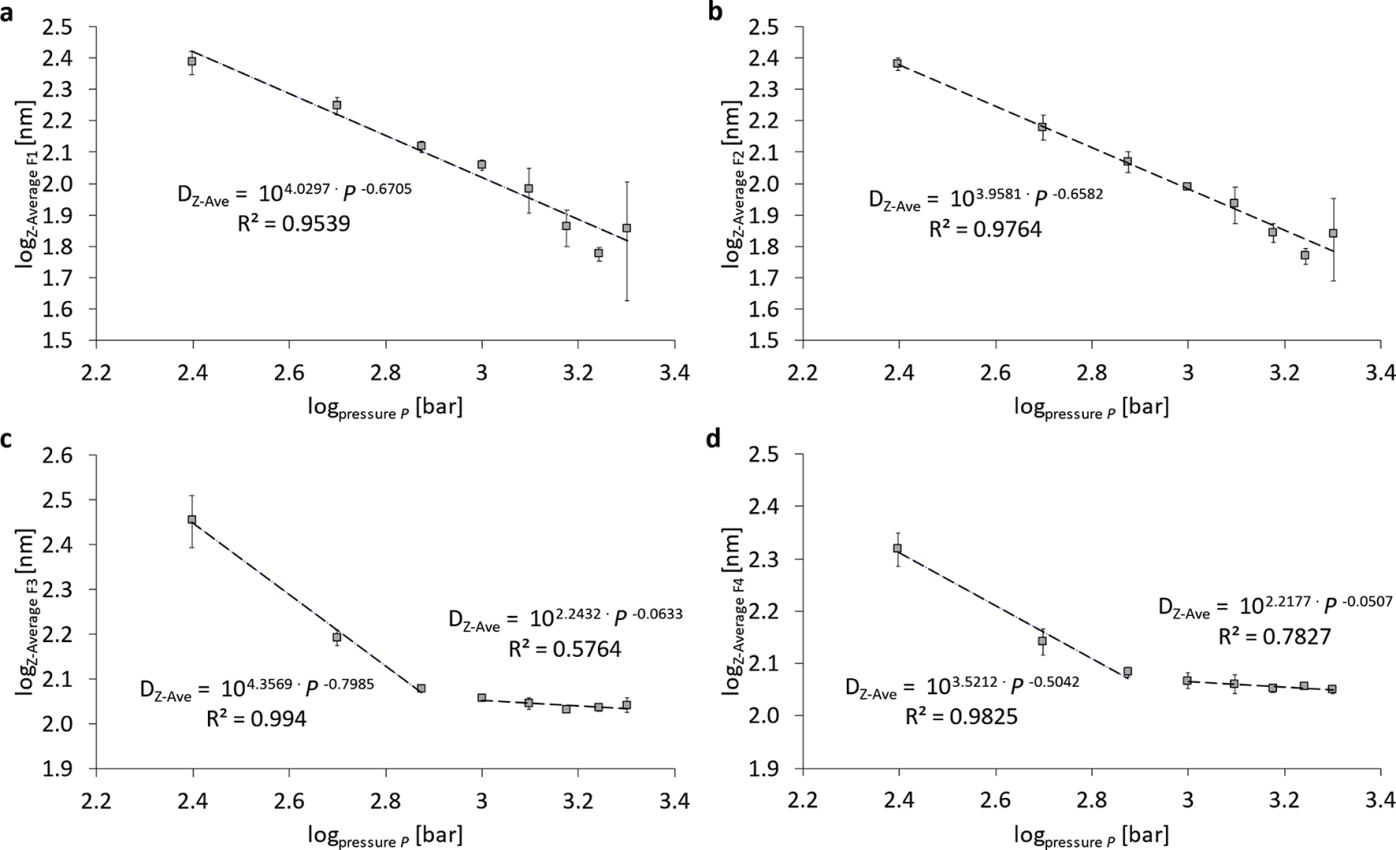
Logarithmic representation of the pressure-dependent particle size (Z-Average) after separation using a sucrose gradient. Representation of the Z-Average of (**a**) fraction 1 (F1), (**b**) fraction 2 (F2), (**c**) fraction 3 (F3) and (**d**) fraction 4 (F4). Nanoemulsions were prepared by 6 cycles of homogenization using the Y- and Z-channel combined in series.

Figure 5 shows the pressure-dependent particle size after separation of the nanoemulsions using a sucrose gradient. There is a power-law relationship between particle size and homogenization pressure for fraction 1 (Fig. 5a) and fraction 2 (Fig. 5b), which exclusively contain liposomes and no PFC emulsion droplets. The liposomes’ size decrease is: Z-Ave ~ *P*^− 0.65^, so $D \approx 1/\sqrt[3]{{P}^{2}}$ with correlation coefficients of the regression R^2^ > 0.95. For both fractions, there is a limit of size miniaturization at 1,750 bar at a size of *D* ≈ 50 nm. For fraction 3 (Fig. 5c) and fraction 4 (Fig. 5d), size miniaturization is effective for *P* ≤ 750 bar with a power-law exponent of -0.8 and − 0.5, respectively. As can be seen, droplet sizes in F3 and F4 are larger than F5 – F7 at *P* = 250 bar, which can only occur in density separation if the density of such relatively large particles is lower in F3 – F4 than in F5 – F7. This is only possible when liposomes are not fully transformed into monolayers or triple layers, but rather adhere in their liposomal conformation at the interface, cf. Ullmann et al. 2023^43^. Therefore, we conclude that liposomes as emulsifiers only adhere to emulsion droplets at the lowest pressures, but do not occur for *P* = 750 bar. For *P* ≥ 1000 bar no effect of the pressure *P* is visible on the Z-averaged droplet sizes with minimum Z-Ave of 107.8 nm ± 1.5 nm for F3 and 112.7 nm ± 1.8 nm for F4. For the fractions F5 to F9, shown in Supplementary Figure S2, the size decrease in the lower pressure range *P* ≤ 750 bar still occurs, but without a clearly correlated power-law in this range and without significant size change for *P* ≥ 1,000. Minimum Z-averaged sizes in the high-pressure range are: 149.6 nm ± 2 nm (F5); 176.4 nm ± 4.88 nm (F6); 179.2 nm ± 3.2 nm (F7) and 194.7 nm ± 16.9 nm (F8), see Figure S2.

**Fig. 6:**
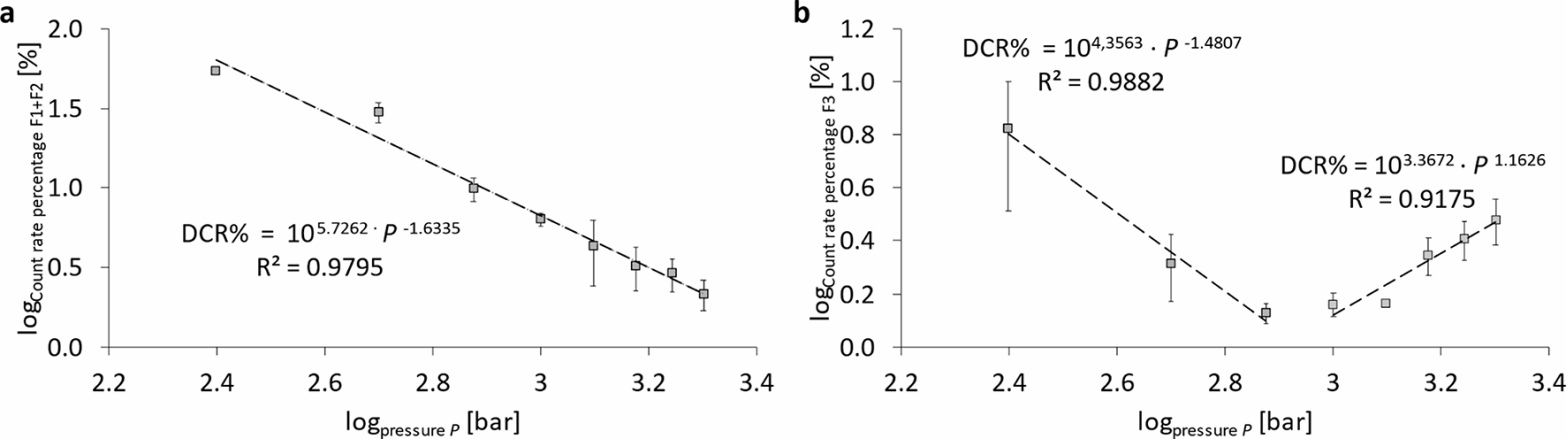
Logarithmic representation of the pressure-dependent count rate percentages of selected fractions after separation using a sucrose gradient. Representation of (**a**) fraction 1 and fraction 2 in total (F1 + F2) and (**b**) fraction 3 (F3). Nanoemulsions were prepared by 6 cycles of homogenization using the Y- and Z-channel combined in series.

In addition to the evaluation of the PFC-droplet size after separation using a sucrose gradient, the assessment of the count rate percentages is relevant and shown in Fig. 6. The maximum Derived Count Rate percentage (DCR%) of the liposomal fractions (F1 + F2) changes from 54.1 ± 2.6 DCR% at 250 bar to 2.2 ± 0.5 DCR% at 2,000 bar. Here, a power-law relationship between pressure and DCR% over the total pressure range can only be seen for fractions 1 plus 2 (Fig. 6a) with a correlation of DCR% ~ *P*^− 1.6335^ ≈ $1/\sqrt[3]{{P}^{5}}$ meaning that the DCR% of the liposomes is approximately reduced to one third upon doubling the pressure. This is the rate by which liposomal phospholipid bilayers are transformed into emulsifying monolayers with increasing pressure. Further, it can be seen that the liposomal fraction is reduced by this power-law regression, irrespective of the changes of the flow regimes as explained for Figs. 3 and 4. This means that the transformation of liposomal bilayers into emulsifying monolayers is triggered by the increasing pressure drop, not by flow rates, shear rates or temperature increases, because the latter have transition plateaus, cf. Figures 3 and 4, while Fig. 6a does not show such a plateau. A pressure-induced mechanism which destabilizes the integrity of liposomes is cavitation which linearly scales with the static pressure and could therefore be the cause of the transformation of phospholipid bilayers into emulsifying monolayers.

For fraction F3, the decrease in DCR% scales with *P*^− 1.4087^ for *P* ≤ 750 bar and with *P*^+ 1.1626^ for *P* ≥ 1,000 bar. The relative decrease of fraction F3 with a minimum at 750 and 1,000 bar is to some extent the consequence of the increase of the DCR in absolute numbers as shown in Fig. 3d which means that at pressures of 500 and 750 bar the total number of emulsion droplets created is strongly increased. However, the decrease in average sizes of F3 and F4 as discussed in Fig. 5 has a decreasing effect on the DCR. The results for fractions F4 to F9 are shown in Figure S3 in the supplement in their pressure dependency. Generally, all fractions F4 – F9 increase monotonously with increasing pressure which is a consequence of the conversion of phospholipid bilayers from liposomes (fractions F1 and F2) into monolayers emulsifying F4 – F9 and leads to an increase in their DCR%. Only the fractions F7 – F9 decrease in their DCR% for pressures *P* ≥ 1,000 bar as a consequence of the high-pressure homogenization. Supplementary Figure S4 shows an overview of the DCR% distribution of all 9 fractions as a series of 8 pie charts for the 8 pressures from 250 to 2,000 bar.

The analysis of the PdI dependency on the pressure and the fraction number is based on the data shown in Figure S5 in the Supplements. As can be seen for all pressures, the lower fraction numbers have higher PdI > 0.15 while the higher fraction numbers have lower PdI ≤ 0.15. For *P* = 250 bar, fractions F1 – F5 are in the high PdI range, for *P* = 500 bar it is F1 – F4 and for *P* = 750 bar it is F1 – F3. In consequence, this means that in order to homogenize the emulsion fractions F3 – F5, a minimum pressure is needed for the break-up of the smallest emulsion droplets while for the liposomal fractions F1 and F2 their PdI ≥ 0.25 for all pressures. In fact, the PdI is even larger after being treated with higher pressures which is a consequence of the fact that liposomes are strongly decreased in their Z-averaged size while the size distribution is obviously not decreased at the same relative rate as the mean. In essence, emulsion droplets can become increasingly homogeneous with increasing pressure while liposomes cannot (see Figure S5), but liposomes decrease stronger in their absolute size (see Figure S2). Moreover, since liposomes have an absolute minimum in their size of about 20 nm^44,45^, liposomes cannot break up into homogeneously sized fragments, but rather preserve a minimum inhomogeneity of their bilayer masses. In contrast, emulsion droplets cannot be broken up into the same droplet sizes as liposomes when using phospholipids as emulsifiers, but the size distributions of emulsions are much narrower than those of liposomes when treated by high-pressure homogenization.

### Experimental investigation of different numbers of homogenization cycles *N*_*c*_ to produce a PFC/W nanoemulsion

**Fig. 7:**
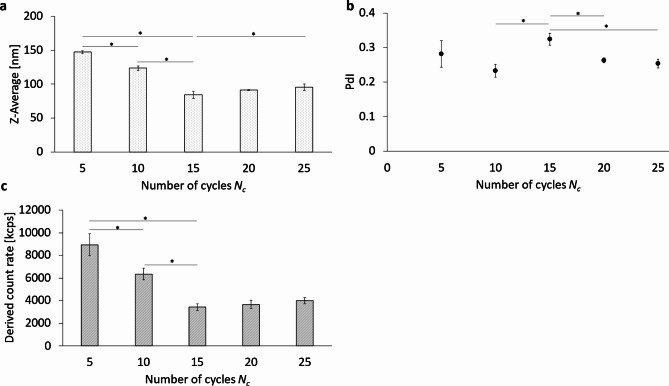
Comparison of different numbers of homogenization cycles *N*_*c*_ using the Y- and Z-channel combined in series. (**a**) The Z-Average (Z-Ave) (**b**) the polydispersity index (PdI) and (**c**) the Derived Count Rate (DCR) of the PFC/W nanoemulsion after 5, 10, 15, 20, or 25 cycles of homogenization at 1,000 bar. The bars and dots represent the mean values with the standard deviation as error bars. A one-way ANOVA followed by a two-sample t-test assuming equal variances was performed at a significance level of *p < 0.05; *n* = 3.

Figure 7 shows the effects of several homogenization cycles on a pre-emulsified sample by serial passage through a Y-channel and downstream Z-channel at a fixed homogenization pressure of 1,000 bar. For this purpose, the Z-Average (Z-Ave), the polydispersity index (PdI) and the Derived Count Rate (DCR) were determined as emulsion characteristics using dynamic light scattering (DLS). For the Z-Ave of the PFC/W nanoemulsion (Fig. 7a), there are significant differences between 5 and 10 cycles, 10 and 15 cycles, 5 and 15 cycles, as well as between 15 and 25 cycles. The smallest Z-averaged particle size is observed at 15 cycles with 84.2 nm ± 5.4 nm. The Z-Ave after 20 cycles is not significantly larger with 91.4 nm ± 4.8 nm. The PdI after 15 cycles of homogenization is significantly higher than at 10, 20, or 25 cycles, but not strongly different at 0.324 ± 0.017 (Fig. 7b). The lowest PdI values can be found after 20 cycles at 0.263 ± 0.007 and 0.253 ± 0.013 after 25 cycles.

Considering the Derived Count Rate (DCR) (Fig. 7c), it is observed that it significantly decreases from 5 to 15 cycles of processing but does not show significant differences from 15 to 25 cycles.

Almost identical results for Z-Ave, PdI and the DCR can be seen after 24 h (Supplement Figure S6) and after 48 h (Supplement Figure S8) of storage at 4 °C respectively.

Figure S7 in the supplement shows the photographic images of the emulsion replicates after the various numbers of homogenization cycles *N*_*c*_ and subsequent storage at 4 °C for 24 h. There is a clear sediment at cycle numbers *N*_*c*_ = 15. Due to the higher density of the perfluorocarbon, it can be concluded that the sediments consist to a large fraction of perfluorocarbons which, however, have very little difference in their refractive index compared with that of water. Therefore, the white appearance of the sediment must be due to the surface coverage of perfluorocarbon by phospholipids.

**Fig. 8:**
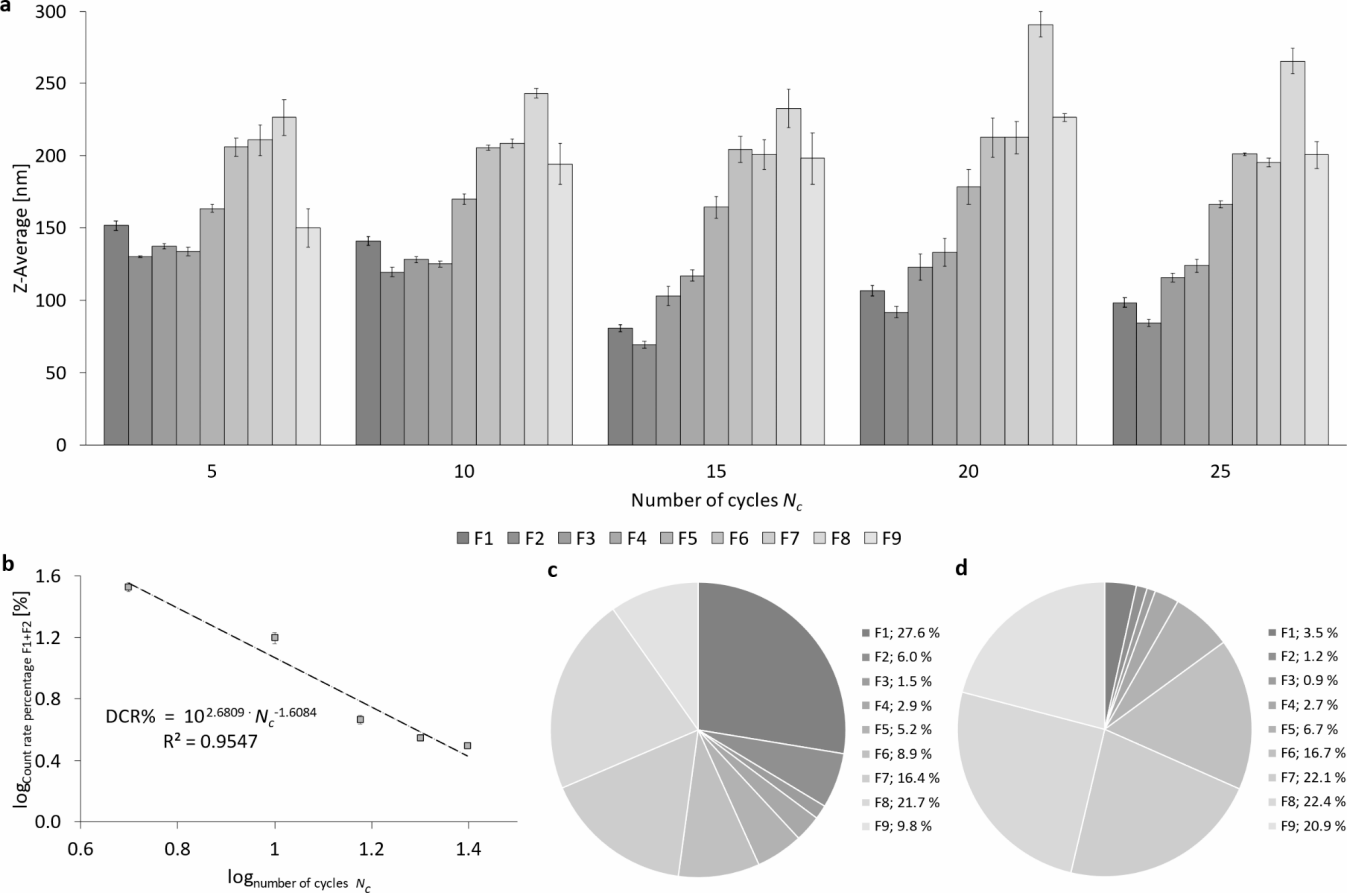
Representation of particle sizes (Z-Average) and DCR% of nanoemulsions after high-pressure homogenization using the Y- and Z-channel combined in series at 1,000 bar with different numbers of cycles *N*_*c*_, showing the fractions F1 – F9 produced by centrifugation in a sucrose gradient (20–60% w/v). (**a**) Representation of Z-Ave of PFC droplets in all fractions from F1 to F9, depending on *N*_*c*_; (**b**) logarithmic representation of DCR% for the combination F1 + F2; (**c**) DCR% of all fractions F1 to F9 of a nanoemulsion homogenized for 5 cycles and (**d**) DCR% of all fractions from F1 to F9 of a nanoemulsion homogenized for 15 cycles.

To evaluate the particle size distributions of PFC nanoemulsion after different numbers of cycles, separation was carried out using a sucrose gradient. The particle size of fractions F1 to F9 as a function of *N*_*c*_ is shown in Fig. 8a. There is an increase in particle size from fraction F3 to F8 for all analyzed cycles, with the particle fractions F1 – F7 being smallest in size after 15 cycles while fractions F8 and F9 show only minor change during the first 15 cycles. After 24 h and also 48 h of storage in the refrigerator at 4 °C, there are no significant differences (Supplementary Figures S9 and S10).

Concerning the count rate percentage of fractions F1 and F2 in total, a power-law relationship exists with the DCR% being proportional to the number of cycles *N*_*c*_, following the regression:

DCR% ~ *N*_*c*_^−1.6084^ (Fig. 8b) with DCR%(F1 + F2) = 33.8 ± 2.2; 4.7 ± 0.3; 3.1 ± 0.1 at *N*_*c*_ = 5; 15; 25, respectively. In general, there is a clear change in the DCR%-distribution with an increase in *N*_*c*_ (Fig. 8c and d). Figure S11 in the supplement shows the logarithmic representation of the DCR% of fractions F1 and F2 in total after 24 h and 48 h of storage at 4 °C. An overview of the DCR%-distributions depending on *N*_*c*_ directly after production and after 24 h and 48 h of storage can be found in Figures S12 to S16 in the supplements.

## Discussion

The most obvious discoveries of our study are the two transitions that can be found when studying the flow of mixed suspensions of liposomes and emulsion droplets covered by phospholipids in high-pressure homogenization channels:

The first of these two transitions occurs in the pressure range between 750 and 1,000 bar, shown in Fig. 3, causing a plateau of the Z-averaged sizes of the particle mixtures and a minimum in the polydispersity index (PdI). This transition is also visible in Fig. 4 as a plateau in both the flow rate and temperature with increasing pressure.

The second transition in the pressure range between 1,500 and 1,750 bar is only visible in Fig. 4, causing again a plateau in the flow rate and temperature with growing pressure.

To unravel the origins of these transitions in the respective pressure ranges, the Reynolds numbers *Re* for the different segments of the Y-channels are shown in Table 1. It can be noticed that transitions from laminar to turbulent flow take place at different pressures for the different segments A, B, and C as shown in Fig. 2. In circular segment A, *Re*_*A*_ is already in its transition range at *P* = 250 bar (*Re*_*A*_ = 2,358). This means that in the flow range with pressures *P* > 250 bar, turbulent flow is formed in the inflow-bearing segment A of the Y-channel. However, turbulence in this segment is not connected to any of the transition phenomena discussed here. In the rectangular cross-section B, the transition to turbulence occurs between 750 and 1,000 bar at *Re*_*B*_ = 2,111 and 2,075, respectively. As has been pointed out in the Results section, transition to turbulence in channel flow with an aspect ratio of α = 0.333 is very likely to occur between *Re*_*crit, theor.*_ = 1,679^37^ and *Re*_*crit, exp.*_ = 2,528^38^. Further, it was already clarified that a relaminarization due to the stagnation flow in the T-shaped crossing is very likely, based on experimental results by Sano & Tamai (2016) and theoretical analyses by Kaewbumrung & Plengsa-Ard (2024)^39,40^. However, the question remains why only the transition to turbulence in section B of the channel should have an influence on the Z-averaged droplet sizes and not those in sections A and C? This question is studied by a comparison of the shear rates in sections A, B and C at the respective transition Reynolds number *Re*_*crit*_. The maximum laminar shear rate ${\left(\partial u/\partial y\right)}_{max}$ for section B is at *Re*_*crit, B*_.(*P =* 750 bar) with ${\left(\partial u/\partial y\right)}_{max}$ = 49,877 s^−1^. In contrast, for section A and C these maximum laminar shear rates are ${\left(\partial u/\partial y\right)}_{A max}$ = 37,726 s^− 1^ and ${\left(\partial u/\partial y\right)}_{B max}$ = 15,277 s^− 1^ at *Re*_*crit, A*_.(*@* 250 bar) and *Re*_*crit, C*_.(*@* 1,500 bar), respectively. This comparison shows that laminar shear stress in the different channels is highest in segment B before the onset of the local transition to turbulence. This comparison clearly explains why segment B is decisive for the minimization of droplet size. The remaining question is why laminar flow has a stronger influence in minimizing droplet sizes compared to turbulent flow? From the pressure exponents it can be seen that the Z-averaged size decreases with *P*^− 1.2333^ in laminar flow, but only with *P*^− 0.7852^ in turbulent flow. For this question, suitable literature on the spatial particle distribution in the rectangular duct cross-section mostly exists for larger particles, i.e. for channel width to particle size ratios < 20 which would refer for our channel (width 600 μm) to particles > 30 μm. For such microparticles, studies in laminar flow by Kazerooni et al. (2017) and in turbulent flow by Fornari et al. (2018) prove differences in partial segregation and formation of inhomogeneous patterns^46,47^. In laminar flow, particles are concentrated in a rectangle with a distance between 40 and 90% from the cetre of the rectangular duct. In contrast, particles concentrate in the corners, the diagonals and at higher concentrations also the centre of turbulent flow in rectangular ducts. As it is also shown by Fornari et al. (2018), shear stresses are minimized in these regions^47^. For particles in the nanometer range with channel width to particle ratios > 2,000, i.e. particles < 300 nm in our case, research is only in its beginnings. Luo et al. (2016) show that in dilute gases, both positive lift forces connected to shear gradients can be found, while also negative lift can be created by temperature differences that inevitably arise in high shear flows^48^. Yuan et al. (2017) describe lift and drag forces on bubbles in nanofluids, considering besides lift forces also the Magnus forces due to particle rotation and interaction of nanoparticles with gas bubbles^49^. From the current status, no clear correlations can be drawn yet between our experimental findings on the different effect strengths of laminar and turbulent flow reducing the averaged sizes of particles. But it can be hypothesized that particles in laminar flow are transported to a high percentage in high-shear zones while particles in turbulent flow migrate to a much higher proportion into lower-shear zones.

It is relevant to discuss the effect strength of high-pressure homogenization on droplet minimization exerted on the different particle fractions F1 – F9 fractionated by the sucrose gradient (20–60% w/v). As it is shown in Fig. 5 and S2, only the liposomal fractions F1 and F2 are reduced in size over the full pressure range with a correlation between Z-averaged size *D* and pressure *P* of *D*(F1; F2) *~ P*^− 0.67^. For fractions F3 and F4, the size decrease only occurs for *P* ≤ 750 bar with correlations of *D*(F3) *~ P*^− 0.8^ and *D*(F4) *~ P*^− 0.5^. All fractions F5 – F9 show much lower power-laws of their decrease in size. These findings allow several conclusions:

- Only liposomes can be broken into smaller vesicles over the full pressure range and at equal burst rate, leading to a correlation of droplet size *D* to pressure of *D*(F1; F2) *~ P*^− 0.67^.
- From the emulsion droplets, only fractions F3 and F4 allow to be considerably reduced in size (with a decreasing rate which scales with *P*^− 0.8^ or *P*^− 0.5^) in the lower pressure range *P* ≤ 750 bar. For all other fractions F5 – F9 the power-law exponent is > – 0.2. Moreover, it is apparent that the Z-Ave of fractions F3 are larger than F4. Density fractionation sorts particles of equal density in ascending order of size with increasing sucrose density. Therefore, such inversion can only occur if the density of F3 is (slightly) smaller than that of F4 which is the case for liposome/emulsion aggregates, i.e. the partial attachment of liposomes to emulsion droplets. This scenario is likely since at the lowest pressure of 250 bar, the relative abundance of liposomes (F1 + F2) is 54.1 DCR% ± 2.6 DCR% (see Figure S3) which means that at this low pressure, break-up of liposomal bilayers to form emulsifying monolayers is yet very incomplete. In contrast, at 750 bar, the relative abundance of liposomes (F1 + F2) is only 9.9 DCR% ± 1.7 DCR% and at 2,000 bar it is 2.2 DCR% ± 0.5 DCR% (Figure S3). This indicates that emulsification at lower pressures *P* < 750 bar is very incomplete due to the scarcity of emulsifier. Only when most liposomes are broken up, an increase in homogenization pressure leads to a weak decrease of the emulsion droplet sizes *D*(F3 – F9) *~ P*^− 0.2^.
- In the higher pressure range *P* ≥ 1,000 bar, the Z-averaged sizes of the fractions F3 – F9 are of the order of 110 nm (F3); 120 nm (F4); 150 nm (F5); 180 nm (F6); 190 nm (F7); 200 nm (F8), see Fig. 5 and S2.
- The decrease in the Derived Count Rate (DCR) of fractions F1 and F2 at a rate of DCR ~ *P*^− 1.63^ shows that doubling the pressure decreases the number of liposomes by 68 DCR%. Correspondingly, the increase of fractions F3 – F9 reflects the break-up of liposomes and their transformation of phospholipid bilayer phases into emulsifying monolayers with growth rates of about 68 DCR% by doubling the pressure, see Figure S3c.
- The polydispersity index PdI for the different fractions F1 – F9 shows a sharp drop from PdI ≥ 0.25 to PdI ≤ 0.1 which occurs for F6 at *P* = 250 bar and is shifted to F3 at *P* = 750 bar, see Figure S5. This fact also supports the scenario that the fractions F3, F4 and F5 also contain some aggregates of liposomes with emulsion droplets at low pressures *P* ≤ 750 bar, which makes these fractions more inhomogeneous in their sizes.
- Figure S5 clearly shows that all PFC nanoemulsion droplets (i.e. fractions F3-F9) have PdI < 0.3 for *P* ≤ 500 bar and PdI < 0.2 for *P* ≥ 750 bar, with many fractions even showing PdI < 0.1. This means that PFC nanoemulsions can be separated by the density gradient method into narrow monodisperse size distributions (PdI < 0.1) or at least in low polydisperse (PdI < 0.2) size distributions. Such narrow size distributions allow to make reliable size quantifications of the different fractions.

In the following, the variation of the number of homogenization cycles *N*_*c*_ is discussed, which sheds more light on the mechanisms of droplet break-up:

- The Z-averaged, non-fractionated particle sizes (Fig. 7) only decrease for the first 15 homogenization cycles (numbers of cycles *N*_*c*_ ≤ 15) from 150 to 80 nm and remains constant for *N*_*c*_ ≥ 15. The following must be taken into account: data with varying *N*_*c*_ are generated at a constant pressure of 1,000 bar while data with varying pressure are taken at *N*_*c*_ = 6 and are therefore not comparable in their absolute numbers, only regarding their trends and mechanisms. Considering also the size distribution after fractionation in Fig. 8a, it can be concluded that additional homogenization cycles for *N*_*c*_ > 15 do not further minimize the sizes of the liposome and emulsion droplet fractions. In contrast, a small increase of the Z-Ave comparing *N*_*c*_ = 15 with 25 as can be seen in Fig. 7a.
- The transformation of the liposome-forming phospholipid bilayers into emulsion monolayers occurs at an equal logarithmic rate of $DCR\sim{N}_{c}^{-1.6}$ between 5 and 25 cycles (at constant pressure of 1,000 bar). Roughly speaking, this means that doubling *N*_*c*_ leads to a decrease of the number of liposomes by 67%.
- There is a coincidence in the effect of either doubling of the pressure *P* exerted on the liposomes or alternatively doubling the *N*_*c*_ which both lead to decrease the DCR% of liposomes by 68% or 67%, respectively. This means that prolonging the shear rates or doubling the shear rates (in the laminar regime where shear rates are proportional to the pressure applied) have the same effect in transforming liposomal bilayers into emulsifying monolayers.
- A closer look at the results in Fig. 7a reveals that an increase in homogenization cycles beyond 15 cycles results in a small, but significant increase in the resulting particle size at 25 homogenization cycles. This can be explained by an “over-processing”, where the emulsion droplet size increases despite a continued energy input due to a high rate of recoalescence of new droplets^1,4,7,50–54^. It is important to note that the emulsification process is always to be considered as a “balance” of two counteracting sub-processes: droplet break-up vs. re-coalescence^3,55,56^. Freshly produced droplets tend to re-coalesce, i.e. fuse due to thermodynamic instability and incomplete coverage of the interfaces with emulsifier molecules. As a result of Brownian molecular motion and the strong turbulence in the emulsifier systems, the emulsion droplets are subjected to strong relative motion, which leads to droplet collisions^57^.

As shown here, repetitive cycles of high-pressure homogenization are only effective for a limited number of cycles *N*_*c*_. For the configuration shown here, 15 cycles are sufficient for the configuration used at a pressure of 1,000 bar. Beyond this threshold, repetitive cycles will not lead to a measurable decrease in the size characteristics of the different particle species and their size fractions. Instead, additional homogenization will progressively lead to increased particle collisions and aggregation effects.

## Conclusion

In conclusion, the pressure-dependent homogenization study of a mixed suspension containing liposomes and PFC-nanoemulsion droplets covered with phospholipids reveals a complex transition from laminar to turbulent flow, significantly impacting particle size and distribution. The transition to turbulence, particularly in specific sections of the interaction chambers, correlates with changes in droplet size, emphasizing the importance of local shear rates. Notably, liposome sizes continuously decrease with increasing pressure, while PFC-droplet sizes only decrease up to 750 bar, stabilizing thereafter. The impact of the number of homogenization cycles also varies between particle types, with a notable recoalescence effect observed after 15 cycles, leading to an increase in droplet size despite further energy input. This behavior underscores the role of thermodynamic stability and emulsifier coverage in influencing particle stability and size during high-pressure homogenization.

## Supplementary Information

Below is the link to the electronic supplementary material.

Supplementary Material 1

## Data Availability

Data will be made available on request.
