# Supplementary material for: Laminar and turbulent flow effects in high-pressure homogenization of liposomes and perfluorocarbon nanoemulsions
Source: Sci Rep. 2024 Nov 13;14:27856. doi: 10.1038/s41598-024-78550-9 (PMC11561284; doi:10.1038/s41598-024-78550-9)
Supplement: Supplementary file 1 — Supplementary Material 1 [file 41598_2024_78550_MOESM1_ESM.docx]

Laminar and turbulent flow effects in high-pressure homogenization of liposomes and perfluorocarbon nanoemulsions

# Larissa J Lubitz, Harden Rieger and Gero Leneweit


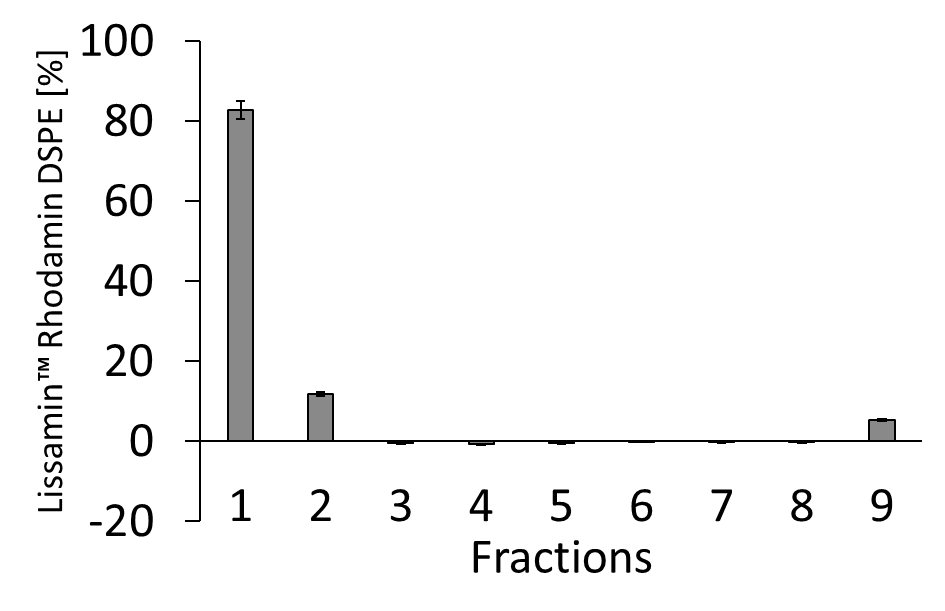


**Supplementary Figure S1:** Identification of fractions with fluorescently labeled liposomes containing 0.1 mol-% of Lissamine™ Rhodamine-DSPE without any perfluorocarbon emulsion. As can be seen, only the fractions F1 and F2 contain liposomes. Fluorescent dye in fraction F9 is a consequence of the aspiration technique by which the fractions are carefully pipetted, but the wall-wetting layer flowing down the centrifugation tube contaminated the final fraction F9. The bars represent the mean ± SD, n = 3.

**Supplementary Table S1:** Comparison of dimensionless numbers characterizing high-pressure homogenization: Reynolds number $Re=\bar{u}_{B}D_{B}/\nu$; Weber number $We=\Delta\rho\bar{u}_{B}^{2}D/\sigma$; Capillary number $Ca=\mu_{PFC}\bar{u}_{B}/\sigma$ with $\bar{u}_{B}$: mean velocity at cross section B; *D_B_*: hydraulic diameter at cross section B; *ν*: kinematic viscosity of the aqueous continuous phase; $\Delta\rho:$ density difference between dispersed phase (PFC) and continuous phase (water) = 1030 kgm^-3^; *D*: Z-averaged . mean droplet diameter depending on homogenization pressure; σ: interfacial tension between water and PFC = 24,5 mN/m.; $\mu_{PFC}$: dynamic viscosity of the dispersed phase (PFC) = 28.4 mPa s.

| **Pressure [bar]** | **Mean velocity in cross section B** ${\bar{\boldsymbol{u}}}_{\boldsymbol{B}}$**[m/s]** | **Mean droplet diameter *D* [nm]** | **Reynolds number *Re*** | **Weber number *We*** | **Capillary number *Ca*** |
| --- | --- | --- | --- | --- | --- |
| 250 | 2.31 | 452 | 882 | 0.10 | 2.74 |
| 500 | 4.03 | 180 | 1,533 | 0.13 | 4.76 |
| 750 | 5.54 | 118 | 2,111 | 0.16 | 6.56 |
| 1,000 | 5.45 | 126 | 2,075 | 0.16 | 6.45 |
| 1,250 | 6.23 | 109 | 2,374 | 0.18 | 7.38 |
| 1,500 | 7.38 | 94 | 2,810 | 0.22 | 8.73 |
| 1,750 | 7.68 | 82 | 2,925 | 0.21 | 9.09 |
| 2,00ß | 9.57 | 74 | 3,647 | 0.29 | 11.33 |


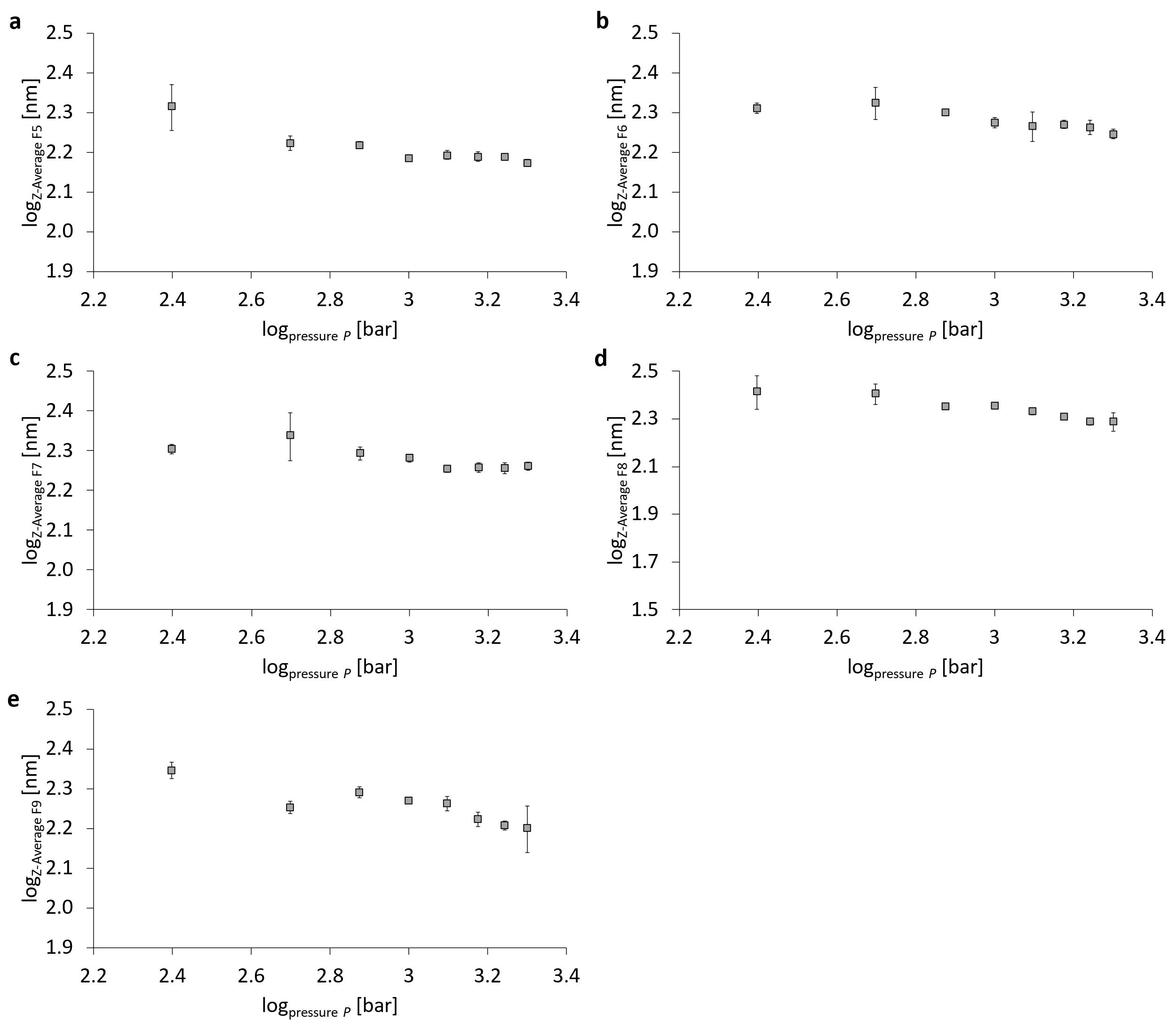


**Supplementary Figure S2:** Logarithmic representation of the pressure-dependent PFC-droplet size (Z-Average) of different fractions after separation using a sucrose gradient. Representation of logarithmic particle size of (a) fraction 5 (F5), (b) fraction 6 (F6), (c) fraction 7 (F7), (d) fraction 8 (F8) and (e) fraction 9 (F9). The dots represent the mean ± SD, n = 3.


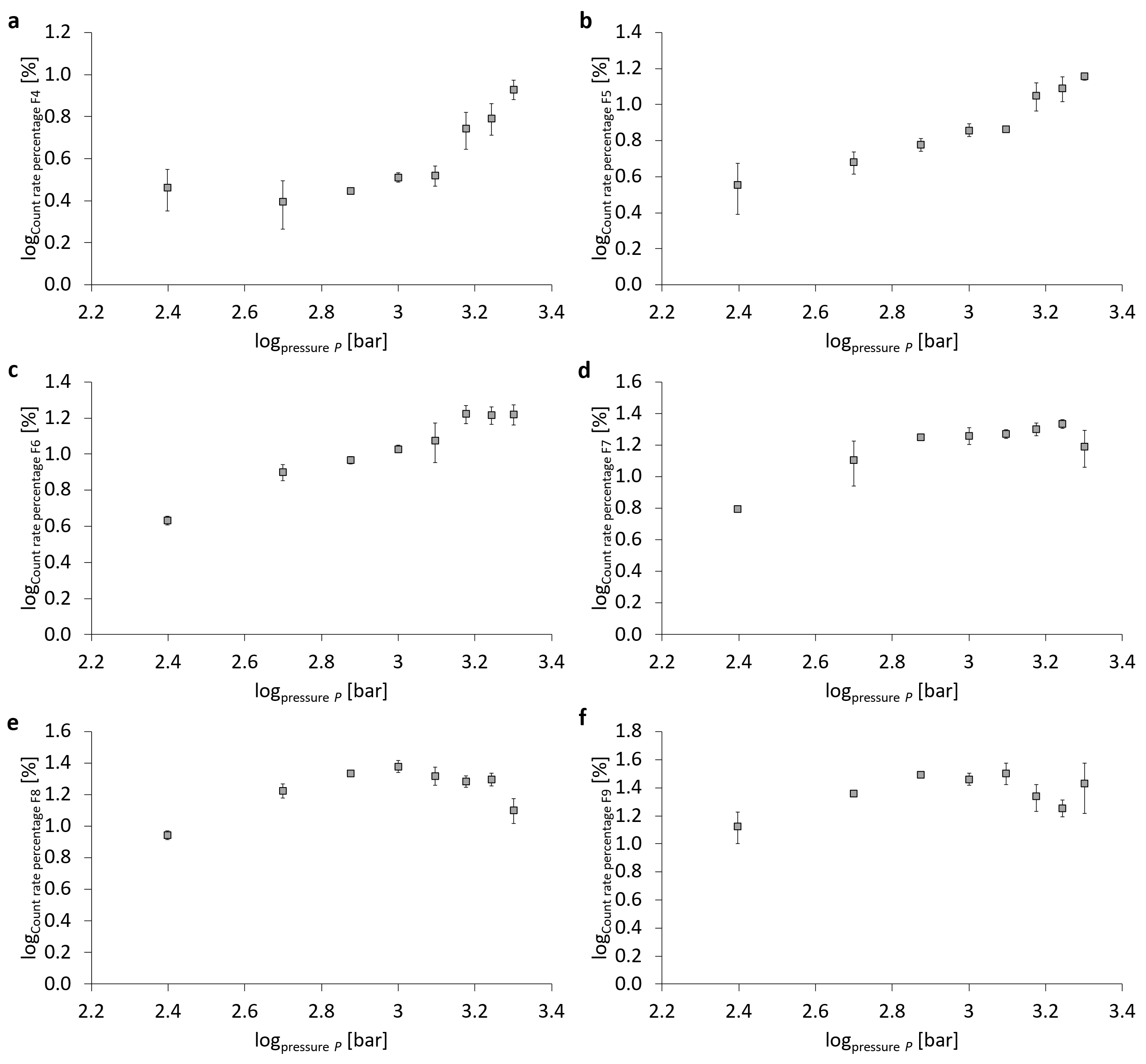


**Supplementary Figure S3:** Logarithmic representation of the pressure-dependent count rate percentage of different fractions after separation using a sucrose gradient. Representation of logarithmic count rate percentage of (a) fraction 4 (F4), (b) fraction 5 (F5), (c) fraction 6 (F6), (d) fraction 7 (F7), (e) fraction 8 (F8) and (f) fraction 9 (F9). The dots represent the mean ± SD, n = 3.


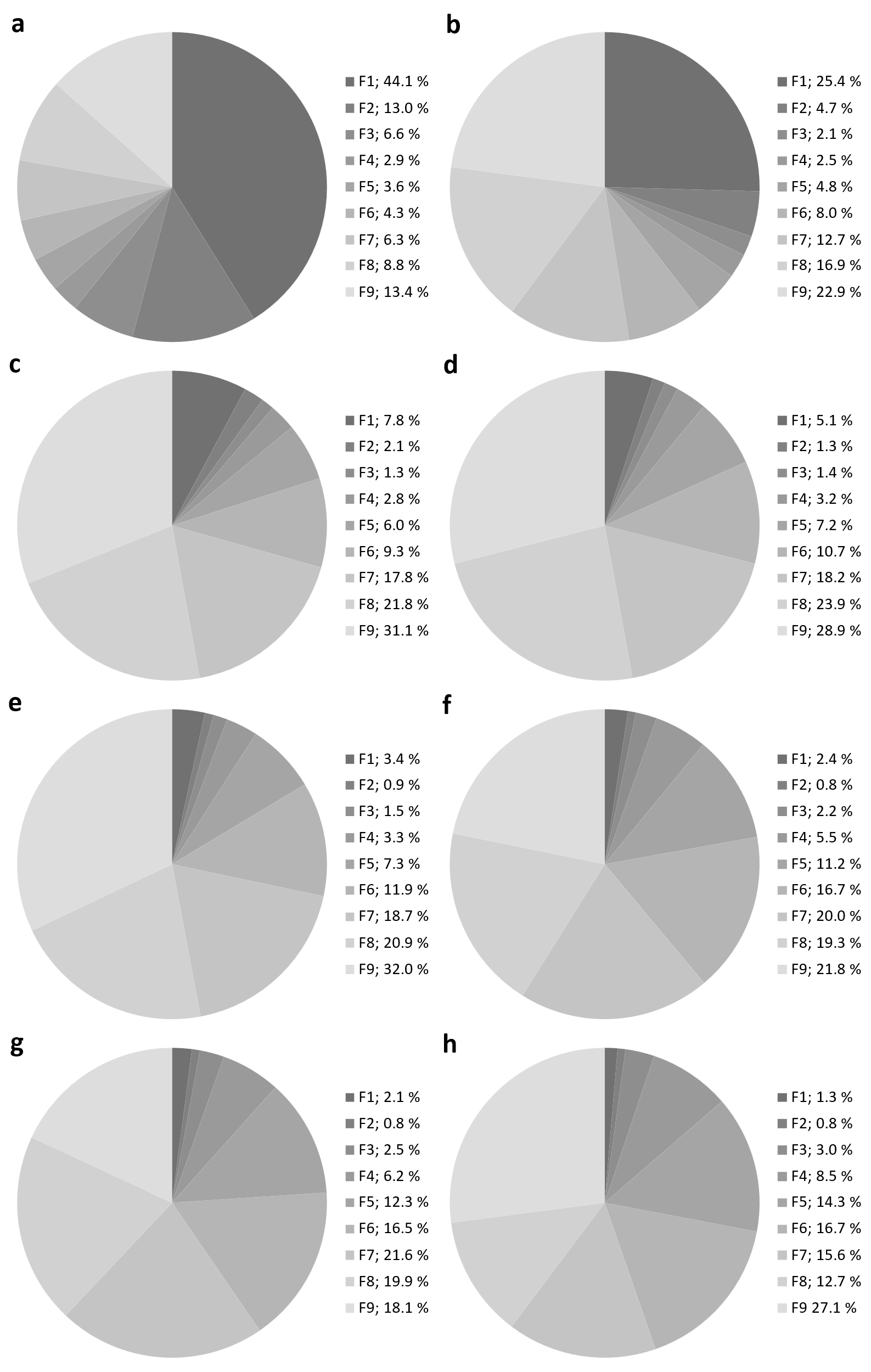


**Supplementary Figure S4:** Pressure-dependent count rate percentages of all fractions after separation using a sucrose gradient. Representation of the count rate percentages at (a) 250 bar, (b) 500 bar, (c) 750 bar, (d) 1,000 bar, (e) 1,250 bar, (f) 1,500 bar; (g) 1,750 bar and (h) 2,000 bar, n = 3.


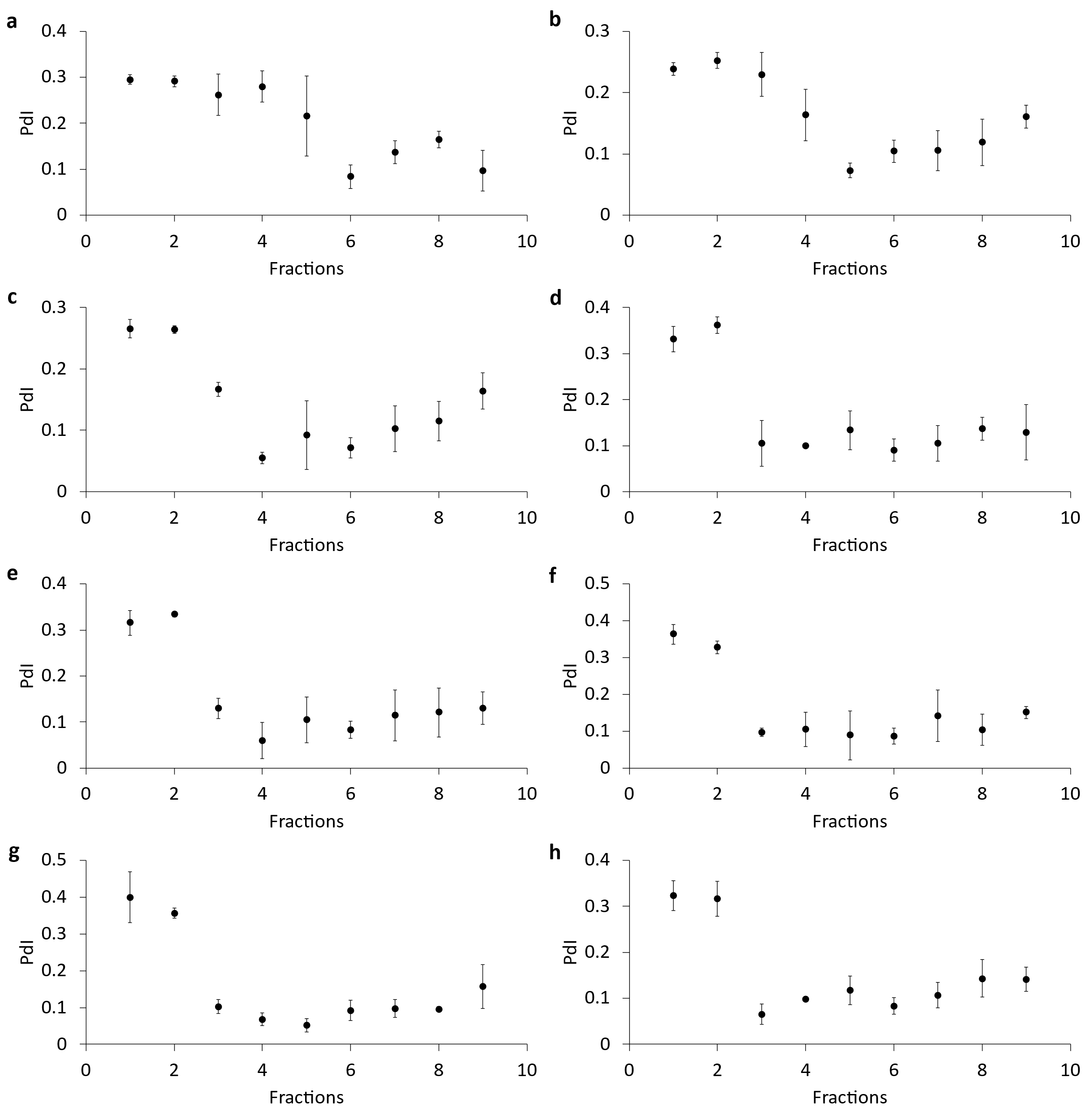


**Supplementary Figure S5:** Pressure-dependent polydispersity indices (PdI) of PFC/W nanoemulsions after separation using a sucrose gradient. Representation of the PdI in all fractions from F1 to F9 for a PFC/W nanoemulsion homogenized at (a) 250 bar, (b) 500 bar, (c) 750 bar, (d) 1,000 bar, (e) 1,250 bar, (f) 1,500 bar, (g) 1,750 bar and (h) 2,000 bar. The dots represent the mean ± SD, n = 3.


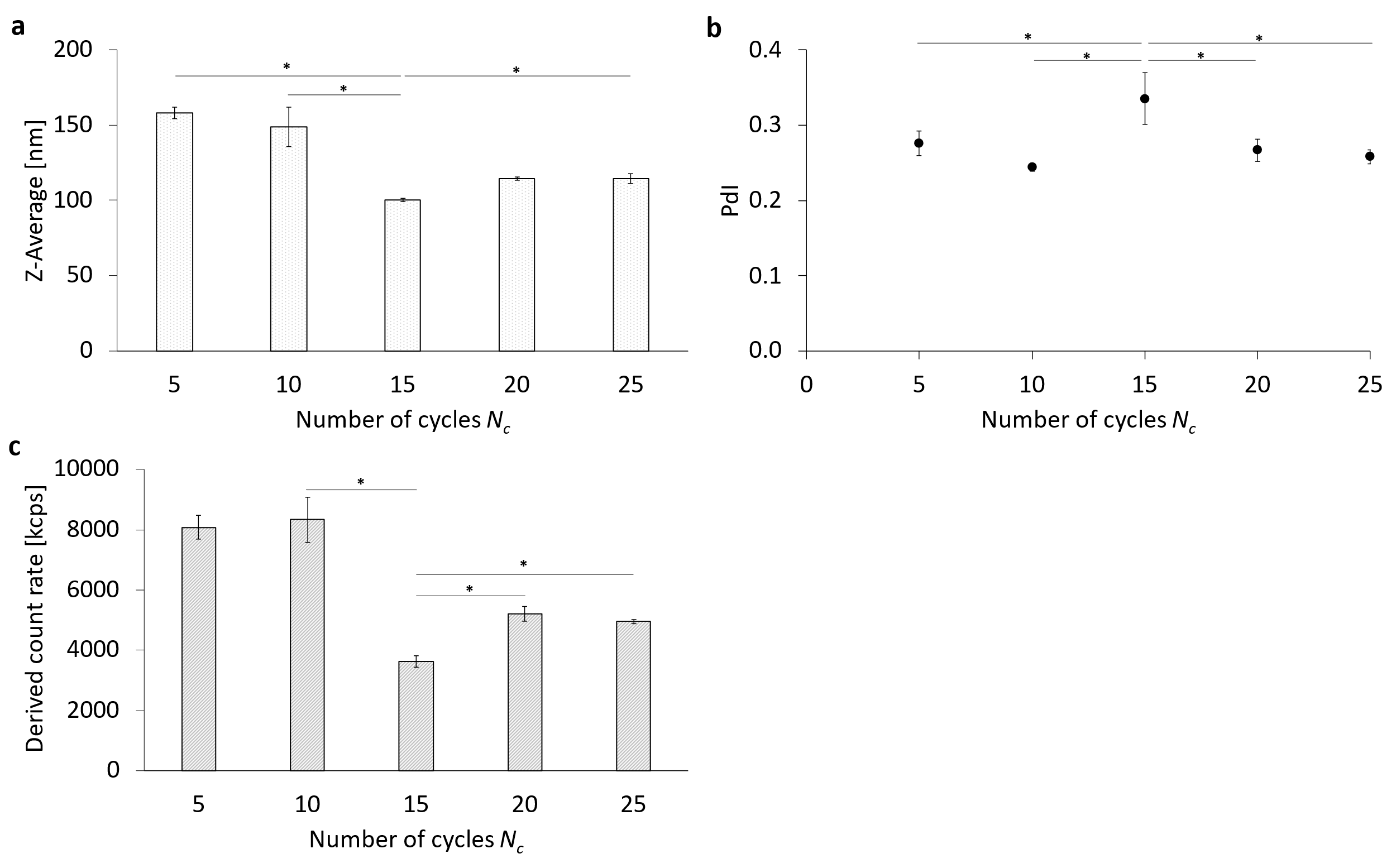


**Supplementary Figure S6**: Comparison of different numbers of homogenization cycles after 24 hours of storage at 4 °C. (a) The Z-Average (Z-Ave) (b) the polydispersity index (PdI) and (c) the derived count rate (DCR) of the PFC/W nanoemulsion after 5, 10, 15, 20, or 25 cycles of homogenization at 1000 bar. The bars or dots represent the mean values with the standard deviation as error bars. A one-way ANOVA followed by a two-sample t-test assuming equal variances was performed at a significance level of *p < 0.05; n = 3. The bars or dots represent the mean ± SD, n = 3.


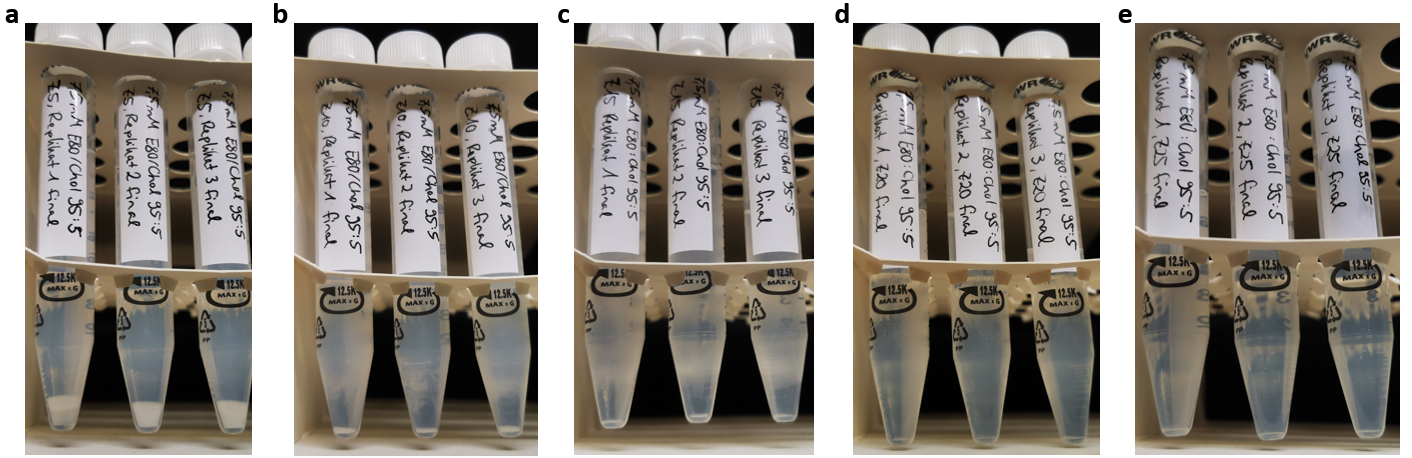


**Supplementary Figure S7:** Photos of the PFC nanoemulsions after different numbers of homogenization cycles after 24 hours of storage time at 4°C. (a) Nanoemulsion after 5 cycles, (b) after 10 cycles, (c) after 15 cycles; (d) after 20 cycles and (e) after 25 cycles of homogenization.


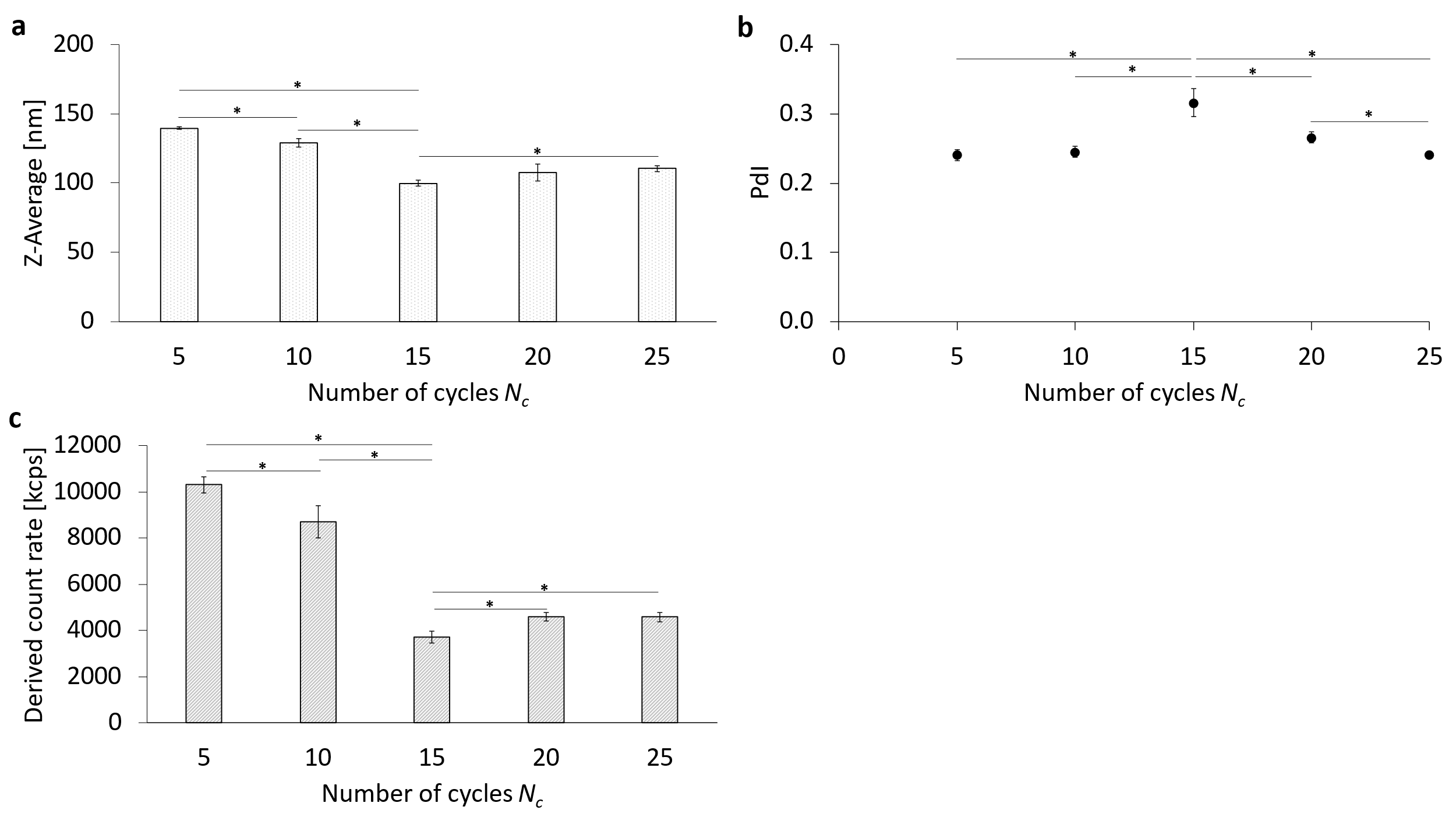


**Supplementary Figure S8**: Comparison of different numbers of homogenization cycles after 48 hours of storage at 4 °C. (a) The Z-Average (Z-Ave) (b) the polydispersity index (PdI) and (c) the derived count rate (DCR) of the PFC/W nanoemulsion after 5, 10, 15, 20, or 25 cycles of homogenization at 1000 bar. The bars or dots represent the mean values with the standard deviation as error bars. A one-way ANOVA followed by a two-sample t-test assuming equal variances was performed at a significance level of *p < 0.05; n = 3. The bars or dots represent the mean ± SD, n = 3.


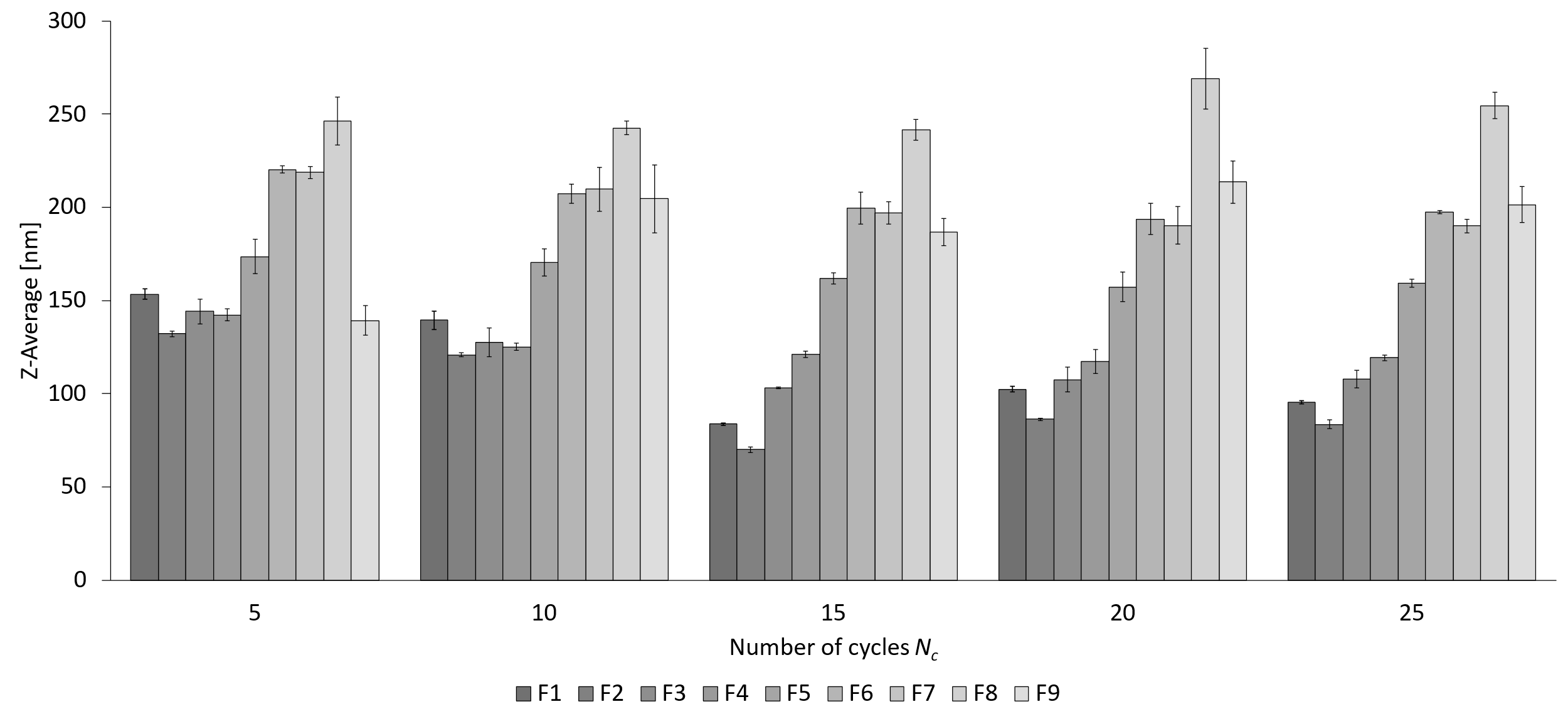


**Supplementary Figure S9:** Representation of cycle number-dependent particle sizes (Z-Average) of all fractions from F1 to F9 after separation using the sucrose gradient after 24 hours of storage at 4 °C. The bars represent the mean ± SD, n = 3.


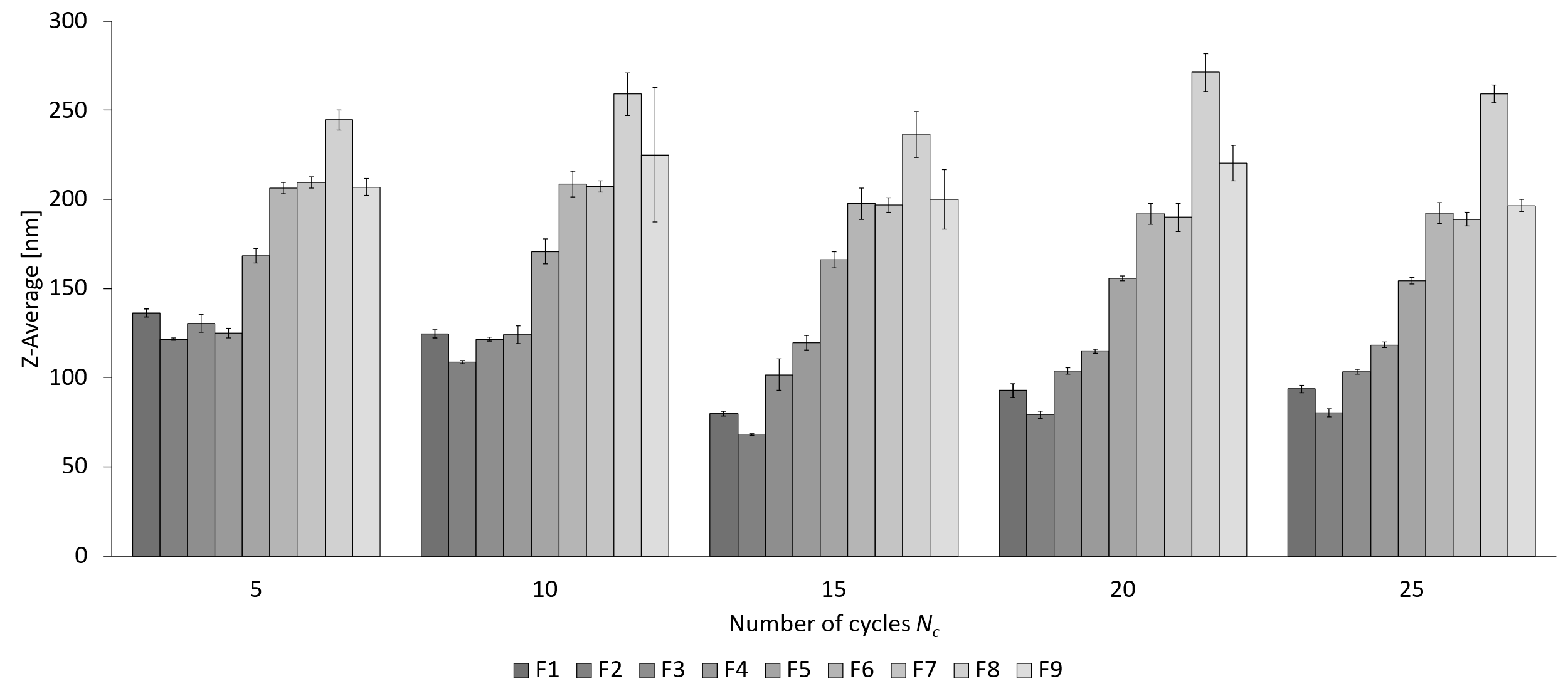


**Supplementary Figure S10:** Representation of cycle number-dependent particle sizes (Z-Average) of all fractions from F1 to F9 after separation using the sucrose gradient after 48 hours of storage at 4 °C. The bars represent the mean ± SD, n = 3.


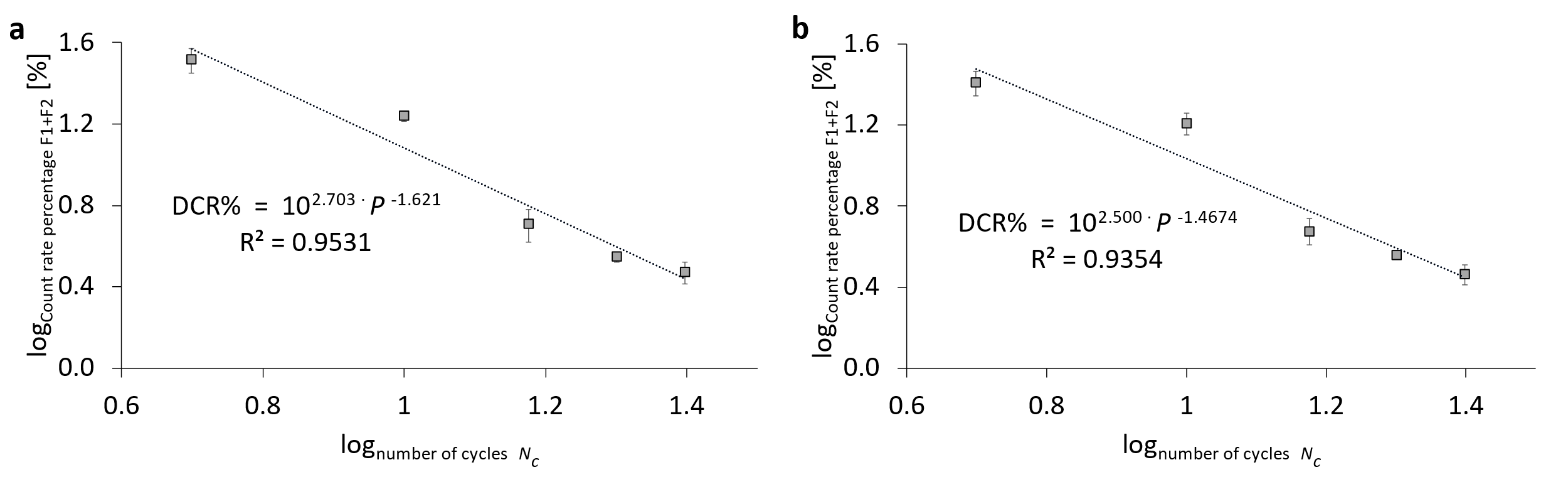


**Supplementary Figure S11**: Logarithmic representation of count rate percentages of fractions 1 and 2 in total (F1+F2) after separation using the sucrose gradient. Representation of count rate percentages of fraction 1 and 2 after (a) 24 hours and (b) 48 hours of storage at 4 °C. The dots represent the mean ± SD, n = 3.


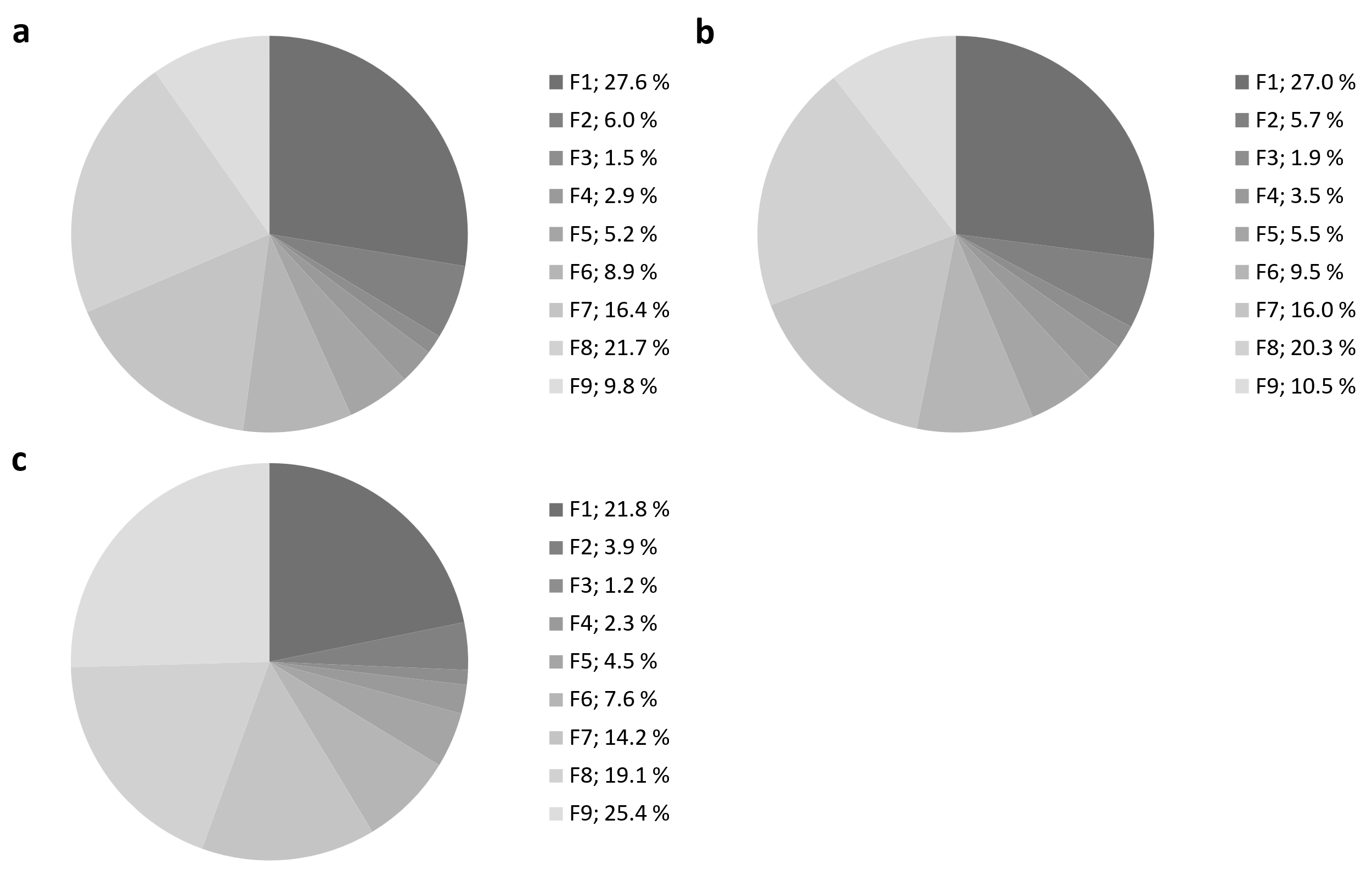


**Supplementary Figure S12:** DCR% of fractions 1 to 9 after separation of a PFC/W-nanoemulsion homogenized for 5 cycles using a sucrose gradient. (a) DCR% of a freshly prepared PFC/W-nanoemulsion, (b) after 24 hours of storage at 4 °C and (c) after 48 hours of storage at 4 °C; n = 3.


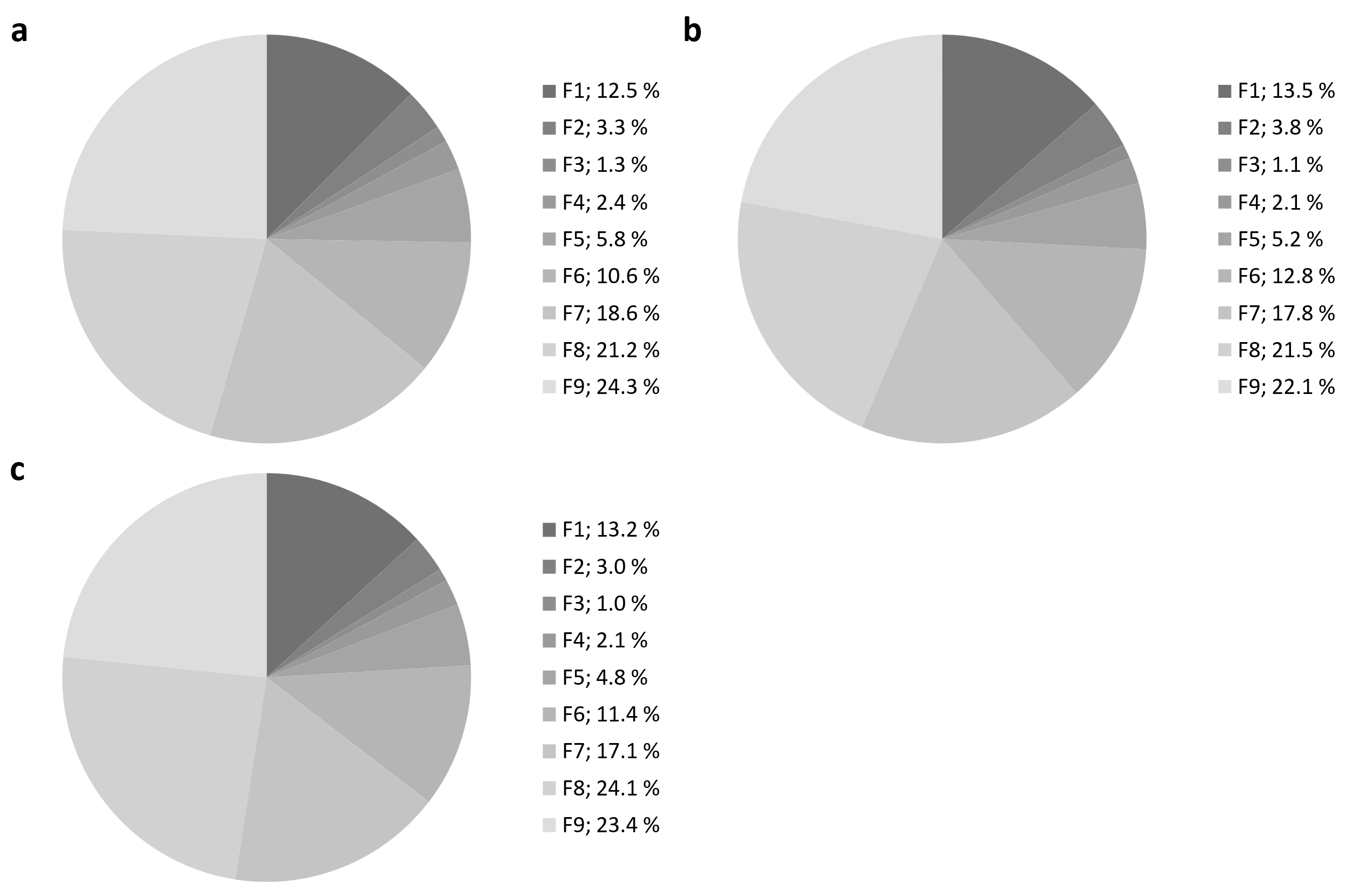


**Supplementary** **Figure S13:** DCR% of fractions 1 to 9 after separation of a PFC/W-nanoemulsion homogenized for 10 cycles using a sucrose gradient. (a) DCR% of a freshly prepared PFC/W-nanoemulsion, (b) after 24 hours of storage at 4 °C and (c) after 48 hours of storage at 4 °C; n = 3.


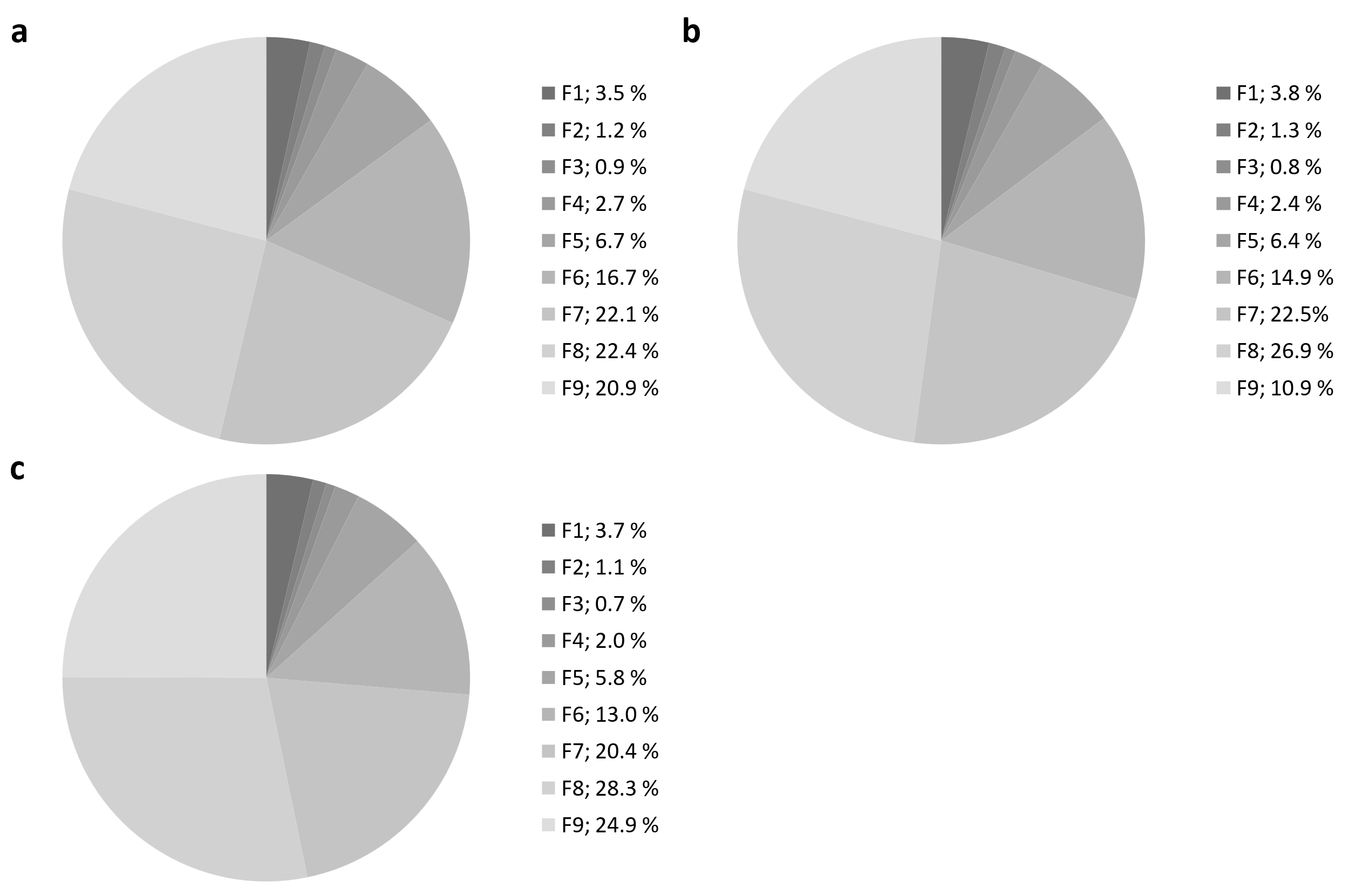


**Supplementary Figure S14:** DCR% of fractions 1 to 9 after separation of a PFC/W-nanoemulsion homogenized for 15 cycles using a sucrose gradient. (a) DCR% of a freshly prepared PFC/W-nanoemulsion, (b) after 24 hours of storage at 4 °C and (c) after 48 hours of storage at 4 °C; n = 3.


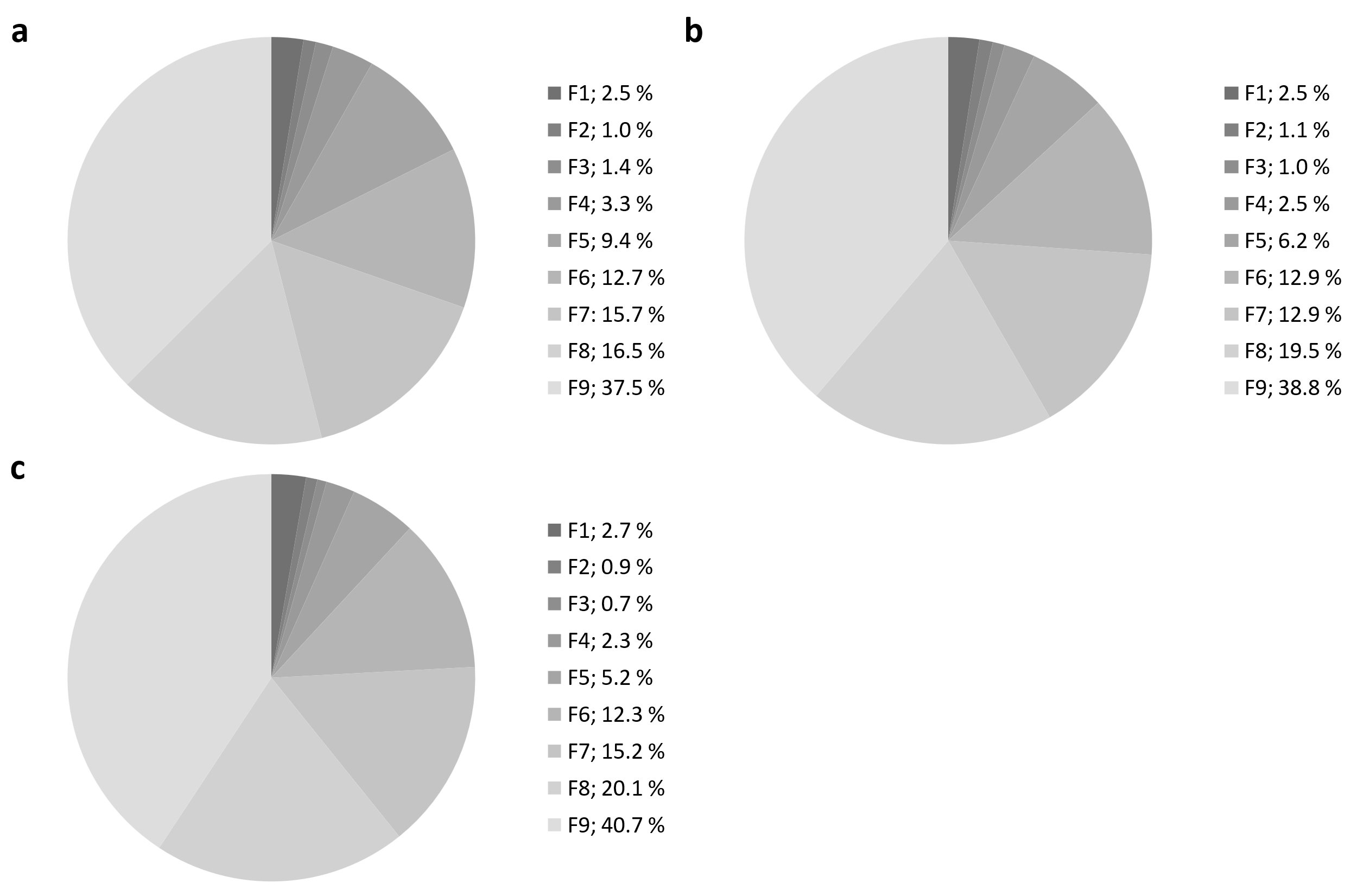


**Supplementary Figure S15:** DCR% of fractions 1 to 9 after separation of a PFC/W-nanoemulsion homogenized for 20 cycles using a sucrose gradient. (a) DCR% of a freshly prepared PFC/W nanoemulsion, (b) after 24 hours of storage at 4 °C and (c) after 48 hours of storage at 4 °C; n = 3.


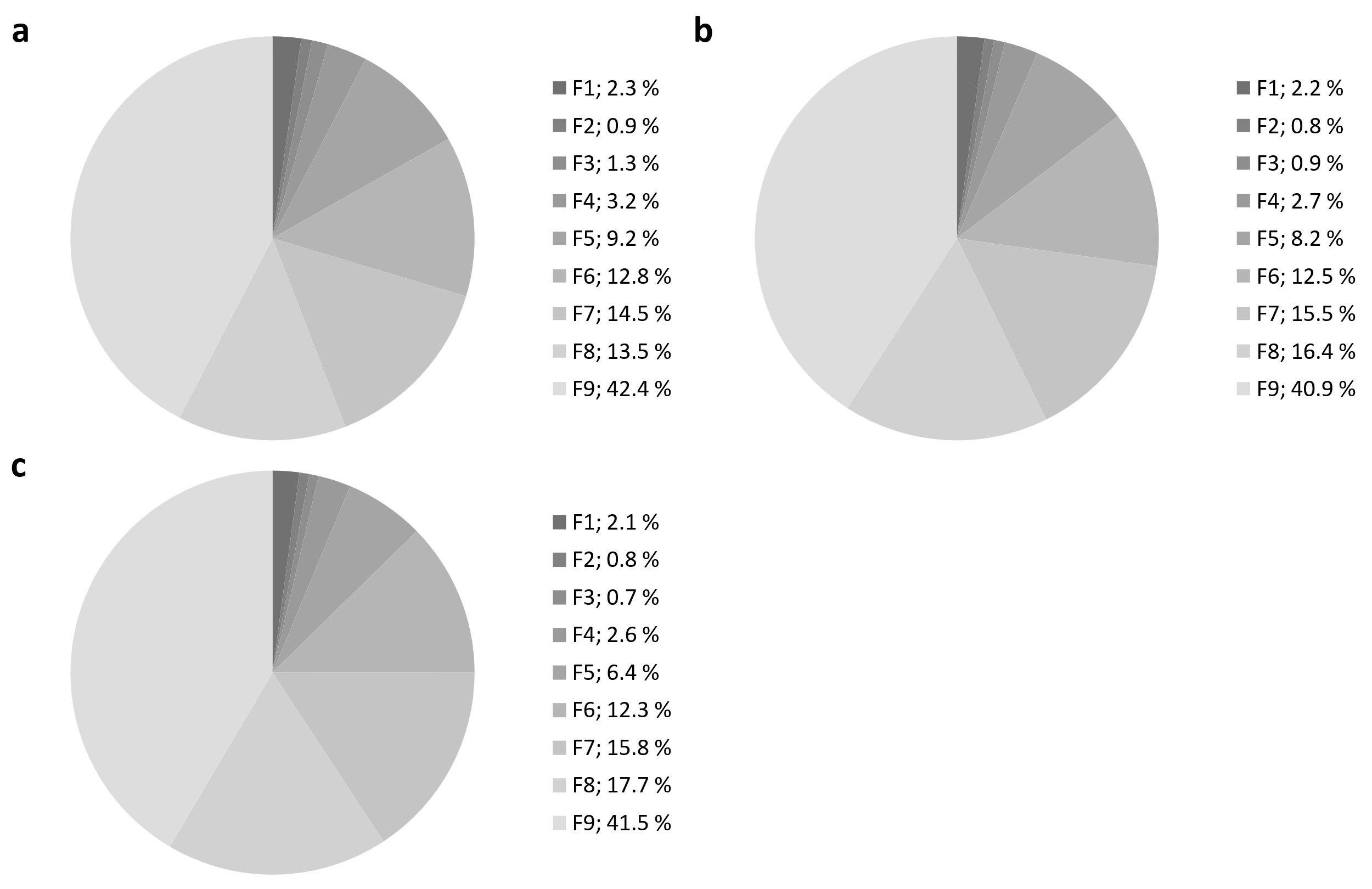


**Supplementary Figure S16:** DCR% of fractions 1 to 9 after separation of a PFC/W nanoemulsion homogenized for 25 cycles using a sucrose gradient. (a) DCR% of a freshly prepared PFC/W nanoemulsion, (b) after 24 hours of storage at 4 °C and (c) after 48 hours of storage at 4 °C; n = 3.
